# Genome-Wide Transcript Profiling Reveals the Coevolution of Plastid Gene Sequences and Transcript Processing Pathways in the Fucoxanthin Dinoflagellate *Karlodinium veneficum*

**DOI:** 10.1093/molbev/msu189

**Published:** 2014-06-12

**Authors:** Elisabeth Richardson, Richard G. Dorrell, Christopher J. Howe

**Affiliations:** ^1^Department of Biochemistry, University of Cambridge, Cambridge, United Kingdom

**Keywords:** dinoflagellate, chloroplast, RNA processing, editing, polyuridylylation, minicircle

## Abstract

Plastids utilize a complex gene expression machinery, which has coevolved with the underlying genome sequence. Relatively, little is known about the genome-wide evolution of transcript processing in algal plastids that have undergone complex endosymbiotic events. We present the first genome-wide study of transcript processing in a plastid acquired through serial endosymbiosis, in the fucoxanthin-containing dinoflagellate *Karlodinium veneficum*. The fucoxanthin dinoflagellate plastid has an extremely divergent genome and utilizes two unusual transcript processing pathways, 3′-poly(U) tail addition and sequence editing, which were acquired following the serial endosymbiosis event. We demonstrate that poly(U) addition and sequence editing are widespread features across the *Karl. veneficum* plastid transcriptome, whereas other dinoflagellate plastid lineages that have arisen through independent serial endosymbiosis events do not utilize either RNA processing pathway. These pathways constrain the effects of divergent sequence evolution in fucoxanthin plastids, for example by correcting mutations in the genomic sequence that would otherwise be deleterious, and are specifically associated with transcripts that encode functional plastid proteins over transcripts of recently generated pseudogenes. These pathways may have additionally facilitated divergent evolution within the *Karl. veneficum* plastid. Transcript editing, for example, has contributed to the evolution of a novel C-terminal sequence extension on the *Karl. veneficum* AtpA protein. We furthermore provide the first complete sequence of an episomal minicircle in a fucoxanthin dinoflagellate plastid, which contains the *dnaK* gene, and gives rise to polyuridylylated and edited transcripts. Our results indicate that RNA processing in fucoxanthin dinoflagellate plastids is evolutionarily dynamic, coevolving with the underlying genome sequence.

## Introduction

Plastid gene expression involves a complex set of transcriptional and posttranscriptional events. Some of the features of plastid gene expression, such as the use of a bacterial RNA polymerase and transcript cleavage, are likely to occur universally across the photosynthetic eukaryotes (Green 2011). Others, such as transcript splicing, sequence editing, and 3′-tail addition, appear to have evolved independently within individual plastid lineages (Asakura et al. 2008; Lange et al. 2009; Fujii and Small 2011), and this may be related to the evolution of the underlying genome sequence. For example, transcript editing in plant plastids, which is predominantly involved in cytosine deamination, is believed to have coevolved with an enrichment in the GC-content of the underlying genome sequence relative to the plastids of related green algae (Fujii and Small 2011).

Until recently, very little was known about the evolution of plastid transcript processing in lineages other than plants. Some of the most important emerging models for studying plastid gene expression in algae are dinoflagellates, and their closest relatives, such as the chromerid species *Chromera velia* and *Vitrella brassicaformis* (Howe et al. 2008; Janouskovec et al. 2013; Dorrell et al. 2014). Dinoflagellates are an evolutionarily diverse group of algae and nonphotosynthetic protists, and have important roles as free-living primary producers, and as symbionts of marine invertebrates such as coral (Howe et al. 2008). The ancestors of all extant dinoflagellates possessed a plastid of red algal origin, of the same endosymbiotic derivation as the plastids found in chromerids, which is retained in species that contain the pigment peridinin (Shalchian-Tabrizi et al. 2006; Janouskovec et al. 2010). The peridinin dinoflagellate plastid has an extremely reduced genome, containing fewer than 15 genes, many of which are highly divergent in sequence, and are located on small, plasmid-like elements termed “minicircles” (Zhang et al. 1999; Howe et al. 2008; Green 2011). Some dinoflagellates have replaced the peridinin-containing plastids with others of a different phylogenetic derivation, through serial endosymbiosis. For example, the fucoxanthin-containing dinoflagellates possess serially acquired plastids derived from haptophyte algae (Takishita et al. 1999; Gabrielsen et al. 2011; Dorrell and Howe 2012). A near-complete plastid genome sequence has been determined for the fucoxanthin dinoflagellate *Karlodinium veneficum*, which retains fewer genes than the plastids of free-living haptophytes, and is highly divergent in sequence (Gabrielsen et al. 2011; Espelund et al. 2012). Other serial endosymbiosis events have occurred in the dinoflagellate genus *Lepidodinium*, which possesses green algal plastids (Takishita et al. 2008; Matsumoto et al. 2011), and the “dinotom” algae, which possess plastids derived from diatoms (Takano et al. 2008; Imanian et al. 2010). Plastid genome sequences have been assembled for the dinotom species *Kryptoperidinium foliaceum* and *Durinskia baltica*, and these retain far more genes, and are less divergent in content than the *Karl. veneficum* plastid genome (Imanian et al. 2010; Gabrielsen et al. 2011).

In addition to possessing very unusual genomes, dinoflagellate plastids utilize a distinctive set of transcript processing pathways. Peridinin dinoflagellate plastid transcripts receive a 3′-terminal poly(U) tail, and this process, while also found in the plastids of chromerid algae, is absent from other plastid lineages, including those of haptophytes and diatoms (Wang and Morse 2006; Janouskovec et al. 2010; Dorrell and Howe 2012). In addition, plastid transcripts in some peridinin dinoflagellates undergo substitutional sequence editing, which can occur on up to one in ten residues in individual transcript sequences and has evolved independently from the much less extensive substitutional editing observed in land plant plastids (Zauner et al. 2004; Fujii and Small 2011; Dorrell and Howe 2012). Recently, we have shown that 3′-terminal poly(U) tail addition and sequence editing occur in plastids of the fucoxanthin dinoflagellate *Karenia mikimotoi* (Dorrell and Howe 2012). Editing has been demonstrated in *Karl. veneficum*, although poly(U) tails have not yet been reported (Jackson et al. 2013). As these pathways are associated with peridinin dinoflagellate plastids and are not found in free-living haptophytes, they are likely to be remnants of the ancestral peridinin-containing plastid symbiosis, applied to the fucoxanthin plastid following its uptake by the dinoflagellate host (Dorrell and Howe 2012). These very recently acquired transcript processing pathways in the highly divergent fucoxanthin dinoflagellate plastid provide a unique opportunity to explore the coevolution of plastid genes and gene expression pathways.

We have surveyed the distribution of poly(U) addition and editing sites across the entire published *Karl. veneficum* plastid genome (Gabrielsen et al. 2011; Espelund et al. 2012). Our study represents the first genome-wide investigation of RNA processing in a plastid acquired by serial endosymbiosis. We demonstrate that almost every gene in the *Karl. veneficum* plastid can give rise to polyuridylylated and edited transcripts, including genes that are not found in the plastid of peridinin dinoflagellates. We demonstrate that the serially acquired plastids in *Lepidodinium* and dinotoms do not utilize either of the RNA processing pathways. We have additionally identified unusual roles for poly(U) addition and editing in highly divergent regions of the *Karl. veneficum* plastid genome. Poly(U) addition may enable the differentiation of functional mRNAs from transcripts of pseudogenes that have arisen through recent genome rearrangements, and editing is associated with fast-evolving sequences and in-frame insertions that have arisen recently in fucoxanthin dinoflagellate plastids. In certain cases, these pathways may have indirectly contributed to the evolution of highly divergent sequences, such as a novel 3′-extension to the *atpA* coding sequence (CDS) that is generated through transcript editing. Most significantly, we present the first complete sequence of an episomal minicircle in a serially acquired dinoflagellate plastid, which has evolved convergently to the minicircles found in peridinin dinoflagellate plastids and gives rise to a polyuridylylated and edited *dnaK* transcript. Our data reveal extensive and complex coevolutionary trends between the plastid genome sequence and transcript processing machinery of fucoxanthin dinoflagellates.

## Results

### Presence of Poly(U) Tails on *Karl. veneficum* Plastid Transcripts

**Fig. 1.**
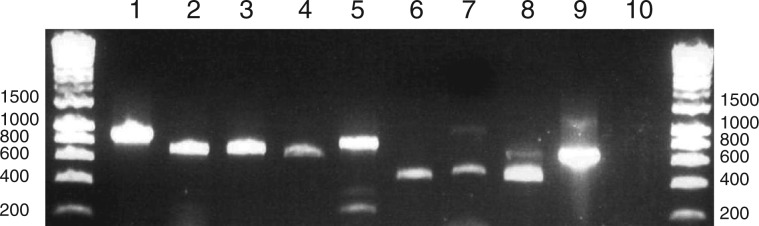
Presence of poly(U) tails in *Karlodinium veneficum* plastid transcripts. The gel photo shows the result of a series of representative oligo-d(A) RT-PCRs for specific transcripts from the *Karl. veneficum* plastid genome. Lanes 1–5: oligo-d(A) RT-PCRs of transcripts that have previously been shown to receive poly(U) tails in *Karenia mikimotoi* (*psbA, psbC, psbD, psaA, rbcL*). Lanes 6 and 7: oligo-d(A) RT-PCRs of representative housekeeping genes (*rpl6, rps5*). Lane 8: oligo-d(A) RT-PCR of the previously unannotated *ORF1*. Lane 9: RT-PCR of *Karl. veneficum psbA* using a cDNA template generated using an internal gene-specific cDNA synthesis and PCR reverse primer, and the same *psbA* forward primer used in lane 1. Lane 10: PCR using the same primers as lane 9, under template negative conditions. The faint secondary band at approximately 1,000 bp in lane 6 corresponds to a dicistronic polyuridylylated *rpl6–rps5* transcript. The secondary bands visible in lanes 5, 8, and 9 were found to be PCR chimeras.

We investigated whether transcripts in the *Karl. veneficum* plastid receive 3′-poly(U) tails, as in the related fucoxanthin dinoflagellate *Kare. mikimotoi* (Dorrell and Howe 2012). As described previously, we performed reverse transcriptions of *Karl. veneficum* total cellular RNA using an oligo-d(A) primer. We then performed PCR using the same oligo-d(A) primer as the PCR reverse primer, and forward primers specific to a representative selection of genes across the *Karl. veneficum* plastid genome (supplementary table S1, Supplementary Material online) (Gabrielsen et al. 2011). These included five photosynthesis genes (*psbA, psbC, psbD, psaA*, and *rbcL*) previously shown to contain poly(U) sites in *Kare. mikimotoi* (fig. 1, lanes 1–5) (Dorrell and Howe 2012). We additionally tested two plastid housekeeping genes (*rpl6* and *rps5*) that have not been investigated in *Kare. mikimotoi* and are not present in peridinin plastid genomes (fig. 1, lanes 6 and 7), and a 603-bp open reading frame (ORF) located in a 1,636-bp previously unannotated region between the *Karl. veneficum chlI* and *psbL* genes that shows no homology to any previously annotated nucleotide or protein sequence, which we henceforth term *ORF1* (fig. 1, lane 8) (Gabrielsen et al. 2011). For each gene tested, we obtained products with the reverse transcriptase polymerase chain reaction (RT-PCR). These products were sequenced and found to correspond to transcripts containing poly(U) sequences located within the 3′-UTR of the gene concerned. These sequences did not correspond to poly(T) tracts in the *Karl. veneficum* plastid genome, and hence are posttranscriptional modifications to the transcript sequence. Our data thus suggest that a wide variety of transcripts in the *Karl. veneficum* plastid receive poly(U) tails, including transcripts of genes that are not found in the plastids of peridinin dinoflagellates.

To confirm that the oligo-d(A) primed RT-PCR products correspond to 3′-terminal transcript poly(U) tails, as opposed to internal sequence insertions, or to artifacts generated by mispriming of the oligo-d(A) primer, we performed RT-PCRs using circular RNA and cDNA and PCR synthesis primers specific to the *Karl. veneficum psbA* and *psbC* genes (supplementary table S1, Supplementary Material online). We have previously employed this technique successfully to confirm the presence of polyuridylylated *psbA* and *psbC* transcripts in *Kare. mikimotoi* (supplementary fig. S1, Supplementary Material online) (Dorrell and Howe 2012). We identified 3′-terminal poly(U) tails on the ends of *Karl. veneficum psbA* and *psbC* transcripts using this approach (supplementary fig. S1, Supplementary Material online). Although we additionally identified nonpolyuridylylated *psbA* transcripts, all of these transcripts terminated at the 3′-end within the CDS and are therefore likely to represent transcript degradation products as opposed to mature transcripts generated by a poly(U)-independent processing pathway (supplementary fig. S1, Supplementary Material online). Our data confirm that poly(U) tails are added to a wide variety of plastid transcripts in *Karl. veneficum*, as with *Kare. mikimotoi*, and suggest that the poly(U) addition pathway was acquired by a common ancestor of extant fucoxanthin dinoflagellates.

### Extent of Poly(U) Addition within the *Karl. veneficum* Plastid

**Fig. 2.**
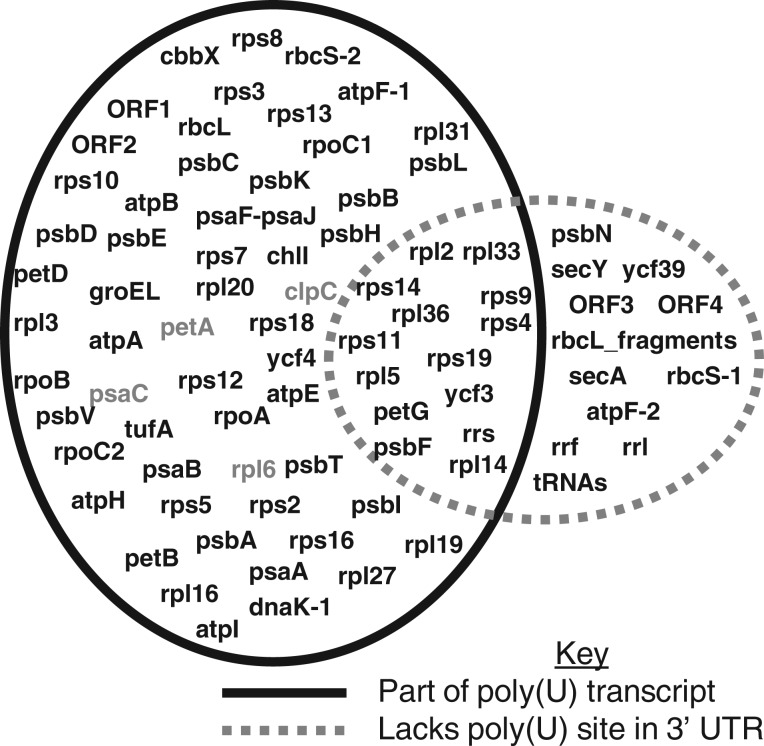
Extent of transcript polyuridylylation across the *Karlodinium veneficum* plastid. The Venn diagram shows the transcript polyuridylylation state of every gene within the *Karl. veneficum* plastid genome. Genes in the overlap sector between the two circles lack directly associated poly(U) sites in their respective 3′-UTR sequences, but can be retrieved as part of polyuridylylated polycistronic transcripts, with the poly(U) site positioned in the 3′-UTR of a downstream gene. The poly(U) tails of genes shaded in gray may be generated by the transcription of genomic poly(T) tracts.

We extended the initial analysis to determine the total extent of transcript polyuridylylation in the *Karl. veneficum* plastid. We performed oligo-d(A) primed RT-PCRs using PCR forward primers for every annotated protein-coding and ribosomal RNA gene within the plastid genome, including previously unannotated *atpE, petG,* and *rps10* genes (supplementary tables S1 and S2, Supplementary Material online). We also tested for the presence of poly(U) tails for 15 predicted tRNA genes in the *Karl. veneficum* plastid genome, and three further predicted ORFs of more than 300 bp length that bear no sequence homology to any previously identified plastid gene (supplementary table S1, Supplementary Material online) (Gabrielsen et al. 2011). We found evidence for widespread polyuridylylation of the *Karl. veneficum* plastid transcriptome, with 54 of the 75 protein-coding genes, and two of the four novel ORFs surveyed possessing poly(U) sites in the associated 3′-UTR (fig. 2 and supplementary table S2, Supplementary Material online). Four of the 56 poly(U) sites observed were positioned within genomic poly(T) tracts (supplementary table S2, Supplementary Material online), so it is possible that they have arisen through primer misannealing. However, the remaining 52 were not and are likely to correspond to posttranscriptional modifications.

For some of the oligo-d(A) RT-PCRs we identified multiple products, consistent with the presence of different polyuridylylated transcripts from a single gene. For example, in the case of *rpl6*, in addition to obtaining PCR products of a size consistent with a monocistronic, polyuridylylated transcript, we observed a secondary higher molecular weight product that was found to correspond to a polyuridylylated dicistronic *rpl6–rps5* transcript (fig. 1, lane 7). We additionally obtained polyuridylylated dicistronic transcripts for 13 of the 21 protein-coding genes that lacked poly(U) sites immediately downstream but were positioned directly upstream of genes that possessed poly(U) sites (supplementary table S2, Supplementary Material online, and fig. 2). This indicates that even genes that do not possess directly adjacent poly(U) sites may give rise to polyuridylylated transcripts.

A small number of the protein-coding genes and unannotated ORFs in the *Karl. veneficum* plastid failed to yield significant products in any oligo-d(A) RT-PCR attempted (supplementary table S2, Supplementary Material online, and fig. 2). In each case, we failed to detect products for each gene even following a nested reamplification of the primary PCR product, with the same oligo-d(A) primer and a second gene-specific primer positioned downstream of the first (supplementary table S1, Supplementary Material online). None of these genes was positioned directly upstream of a gene in the same transcriptional orientation that possessed a poly(U) site, suggesting that they are unlikely to give rise to polycistronic polyuridylylated transcripts (supplementary fig. S2 and table S2, Supplementary Material online). We amplified transcript sequences for these genes, using cDNA synthesis primers internal to the CDS, and these were not completely identical to the underlying genomic sequence, consistent with transcript editing (supplementary tables S1 and S2, Supplementary Material online). We similarly could not identify products in oligo-d(A) RT-PCRs using primers specific to any of the ribosomal RNA subunits or tRNA genes although we identified a tricistronic polyuridylylated *rrs-petG-atpF-1* transcript (fig.2 and supplementary table S1, Supplementary Material online). We generated transcript sequences for all three ribosomal subunits (5.8S, 16S, and 23S rRNA), and could detect low levels of editing in each case (supplementary tables S1 and S2, Supplementary Material online). Our data indicate that the poly(U) addition and editing machinery have been co-opted to recognize almost every gene in the *Karl. veneficum* plastid.

### Location of Poly(U) Sites

We wished to determine what sequence features were associated with the presence of poly(U) sites in the *Karl. veneficum* plastid genome. In chromerid algae, poly(U) addition is biased toward genes encoding proteins that function in photosynthesis (Dorrell et al. 2014). Although photosynthesis genes in the *Karl. veneficum* plastid are more likely to possess an associated poly(U) site than housekeeping genes, the association is not statistically significant (chi-squared, *P* = 0.07) (fig. 2 and supplementary table S2, Supplementary Material online). We additionally compared the gene order of the *Karl. veneficum* genome with those of free-living haptophyte species, and could not identify a consistent relationship between the absence of a poly(U) site, and inferred recombination events (supplementary table S3, Supplementary Material online). Our data therefore indicate that gene function and genome rearrangements are unlikely to be the only factors that determine the distribution of poly(U) sites across the *Karl. veneficum* plastid.

The poly(U) sites within the *Karl. veneficum* plastid are typically positioned close to the 3′-end of the CDS, with an average 3′-UTR length of only 30 bp (supplementary table S2, Supplementary Material online). We looked for conserved primary sequence motifs, changes in GC and purine/pyrimidine content, and predicted RNA secondary structures in the 3′-UTR sequences of each gene, extending 100 bp downstream of each poly(U) site. We could not identify any sequence features that were significantly associated with the presence of a poly(U) site. Several of the poly(U) sites, however, were located within the CDS of the downstream gene (supplementary table S2, Supplementary Material online). Most dramatically, within the ten-gene ribosomal protein operon extending from *rpl3* through to *rps5*, we identified four genes (*rpl3, rpl16, rps8,* and *rpl6*) where the poly(U) site overlaps with the downstream CDS, whereas we only found one poly(U) site within a 3′-UTR sequence, associated with *rps5* (supplementary fig. S3, Supplementary Material online) (Gabrielsen et al. 2011). Using a forward primer specific to *rpl2,* we additionally detected a poly(U) site located 296 bp within the *rpl2* CDS although we could not identify this site using a forward primer specific to the upstream *rpl3* gene (supplementary fig. S3, Supplementary Material online). The poly(U) sites located internal to gene sequences might be associated with alternative end processing events as their formation would prevent transcripts of specific genes being produced from polycistronic precursors. Overall, our data suggest that instead of poly(U) addition being associated with common sequence motifs or specific genes, poly(U) sites are highly sequence-specific. The formation of specific poly(U) sites might influence other events in plastid transcript processing.

### Differential Recognition of Pseudogenes by the *Karl. veneficum* Plastid Transcript Processing Machinery

It has been demonstrated that poly(U) addition discriminates between paralogous copies of genes in the *C. velia* plastid (Dorrell et al. 2014). Transcripts of the *C. velia atpH-1* gene, which are abundant, receive a poly(U) tail, whereas transcripts of the *atpH-2* gene, which appears to be a pseudogene, do not (Janouskovec et al. 2013; Dorrell et al. 2014). Several of the genes in the *Karl. veneficum* plastid are present in multiple copies, some of which appear to be functional, whereas others are likely to be pseudogenes (Gabrielsen et al. 2011). For example, two copies of the *rbcS* gene are present: *rbcS-2*, which is likely to encode a functional protein, and *rbcS-1*, which contains an in-frame insertion within the region encoding the βC-βD loop domain of the rubisco small subunit, that if expressed would be likely to interfere with its function (supplementary fig. S4*A*, Supplementary Material online) (Larson et al. 1997; Li et al. 2005). Similarly, we identified two copies of the *atpF* gene: a previously annotated gene (*atpF-1*) and a previously unannotated pseudogene (*atpF-2*)*,* positioned downstream of and in reverse orientation to *psbB,* which contains an internal frame-shift sequence deletion that would prevent the translation of the complete protein sequence (supplementary fig. S4*B* and table S2, Supplementary Material online).

**Fig. 3.**
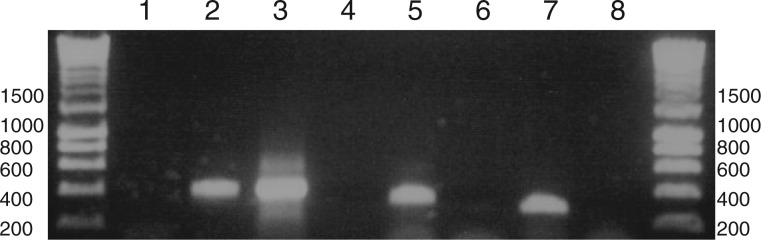
Specific addition of poly(U) tails to transcripts of functional gene paralogs in the *Karlodinium veneficum* plastid. This gel photo shows the result of a series of RT-PCRs to identify whether transcripts of functional and pseudogenic copies of the *rbcS* and *atpF* genes in the *Karl. veneficum* plastid receive poly(U) tails. Lanes 1 and 2: oligo-d(A) RT-PCR of *rbcS-1* (pseudogene) and *rbcS-2* (functional). Lanes 3 and 4: RT-PCR of *rbcS-1* with a gene-specific internal cDNA synthesis primer under template positive (lane 3) and negative (lane 4) conditions. Lanes 5 and 6: oligo-d(A) RT-PCR of *atpF-1* (highly divergent gene) and the *atpF-2* nonfunctional sequence between *rps16* and *psbB*. Lanes 7 and 8: RT-PCR of the *atpF-2* region with a gene-specific cDNA synthesis primer under template positive (lane 7) and negative (lane 8) conditions.

We wished to determine whether transcripts of the *rbcS-1* and *atpF-2* pseudogenes receive poly(U) tails and are edited. We could detect polyuridylylated *rbcS-2* and *atpF-1* transcripts by oligo-d(A) primed RT-PCR, using PCR forward primers specific to each sequence (fig. 3, lanes 2 and 5), but could not detect polyuridylylated *rbcS-1* and *atpF-2* transcripts through the same approach (fig. 3, lanes 1 and 6). We could amplify nonpolyuridylylated *rbcS-1* and *atpF-2* transcript sequences by performing RT-PCRs against cDNA synthesis primers specific to each gene (fig. 3, lanes 3–4, 7–8). We sequenced the products of these RT-PCRs, and confirmed the presence of the in-frame insertion in *rbcS-1* and the frame-shift deletion in *atpF-2* (supplementary fig. S4, Supplementary Material online). We could not identify any editing within the *atpF-2* transcript, and detected only one editing event on the *rbcS-1* transcript, which is significantly fewer than the 15 editing events observed over the same region of the *rbcS-2* transcript sequence (supplementary table S2, Supplementary Material online; binomial test, P< E-05). Our data indicate that poly(U) addition and editing are preferentially associated with functional genes in the *Karl. veneficum* plastid.

### Global Trends in Editing across the *Karl. veneficum* Plastid Transcriptome

Recently, Jackson et al. (2013) have profiled editing events in the *Karl. veneficum* plastid by comparing transcript and genomic sequences for regions of 14 different genes. Four different forms of editing were observed, all of which were transitions, consisting predominantly of A to G and U to C editing events, as well as small numbers of G to A and C to U conversions (Jackson et al. 2013). Across our entire data set, we found evidence for extensive sequence editing (table 1 and supplementary table S2, Supplementary Material online). Approximately 4.3% of sites in our transcript sequences were edited, slightly higher than previous estimates (Jackson et al. 2013). For some genes, we detected higher frequencies of editing, extending to 14% of positions for the *Karl. veneficum psbD* gene, and 24% of residues in the highly divergent *petG* sequence (supplementary table S2, Supplementary Material online). Editing sites were situated predominantly within gene sequences although we detected a low level of editing (1.6%) in polyuridylylated transcript 3′-UTR sequences (supplementary table S2, Supplementary Material online), as previously seen in *Kare. mikimotoi* (Dorrell and Howe 2012). Many (88%) of the editing events lead to an increase in transcript GC-content, consistent with previous studies (Dorrell and Howe 2012; Jackson et al. 2013) (table 1). Although the majority (96%) of editing events observed were transition events, we detected seven different transversion events at low levels in the *Karl. veneficum* transcriptome, similar to our previous observations in *Kare. mikimotoi* (table 1) (Dorrell and Howe 2012).

**Table 1.** Total Editing Events from the Characterized Plastid Transcriptomes of *Karenia mikimotoi* and *Karlodinium veneficum*.

Overview	*Karenia*	*Karlodinium*—Jackson	*Karlodinium*—Extended
Sequence length	5,473	7,373	36,084
A-C	26	0	15
A-G	59	131	789
A-U	0	0	16
C-A	1	0	8
C-G	0	0	4
C-U	17	8	49
G-A	15	15	99
G-C	24	0	8
G-U	0	0	1
U-A	0	0	11
U-C	116	67	540
U-G	2	0	11
Total	260	221	1,539
% Bases edited	4.75	2.86	4.27
% Transitions	79.6	100	96
% Transversions	20.4	0	4
% GC-enrich	78.1	89.6	88
% GC-deplete	12.7	10.4	10
% GC-neutral	9.2	0	1.9
% Nonsynonymous	58.5	95	87.1
% Synonymous	41.5	5	12.9

Note.—The total editing events observed across 36,084-bp *Karlodinium veneficum* plastid transcript sequence in this study are profiled, alongside previous surveys of *Karl. veneficum* (Jackson et al. 2013), and of the related fucoxanthin dinoflagellate *Kare. mikimotoi* (Dorrell and Howe 2012).

Most (87%) of the editing events in the *Karl. veneficum* plastid are predicted to have nonsynonymous effects on the corresponding protein sequence (table 1). Some of these editing events may be required for the correct function of the encoded protein. For example, 11 of the genes in the *Karl. veneficum* plastid contain premature in-frame termination codons, which would prevent the translation of the complete protein sequence. Correction of premature termination codons through editing has previously been reported for *Karl. veneficum rpoB, rps13, psaA* and *secY* transcripts, and *psaA* in *Kare. mikimotoi* (Dorrell and Howe 2012; Jackson et al. 2013). We confirmed that all of the premature termination codons in the *Karl. veneficum* genome are removed from the corresponding polyuridylylated transcript sequences by editing (supplementary table S4, Supplementary Material online). Consistent with previous reports, we also found that edited *Karl. veneficum* transcripts show an increase in sequence similarity, relative to the genomic sequence, to the corresponding sequences from the haptophytes *Emiliania huxleyi* and *Phaeocystis globosa* (supplementary table S4, Supplementary Material online) (Jackson et al. 2013). Editing in the *Karl. veneficum* plastid therefore appears to reduce the effects of divergent mutations on plastid protein sequence.

### Editing of Fast-Evolving Sequences in the *Karl. veneficum* Plastid

Not all of the nonsynonymous editing events observed within the *Karl. veneficum* plastid have readily inferred effects on plastid protein function. Across our entire data set, we found that although more than one in ten codons undergo a nonsynonymous change due to editing, this only leads to a net increase of 1.6% in sequence conservation between the *Karl. veneficum* and haptophyte protein sequences (supplementary table S4, Supplementary Material online). The other editing events may have selectively neutral or disadvantageous effects, or affect sequences that are not found in free-living haptophytes. Notably, many of the genes in the *Karl. veneficum* plastid genome contain novel sequence insertions, or fast-diverging regions that bear no homology to haptophyte sequences (Gabrielsen et al. 2011). We hypothesized that editing events that do not increase sequence conservation with haptophyte orthologs might instead affect sequences unique to the *Karl. veneficum* plastid.

**Fig. 4.**
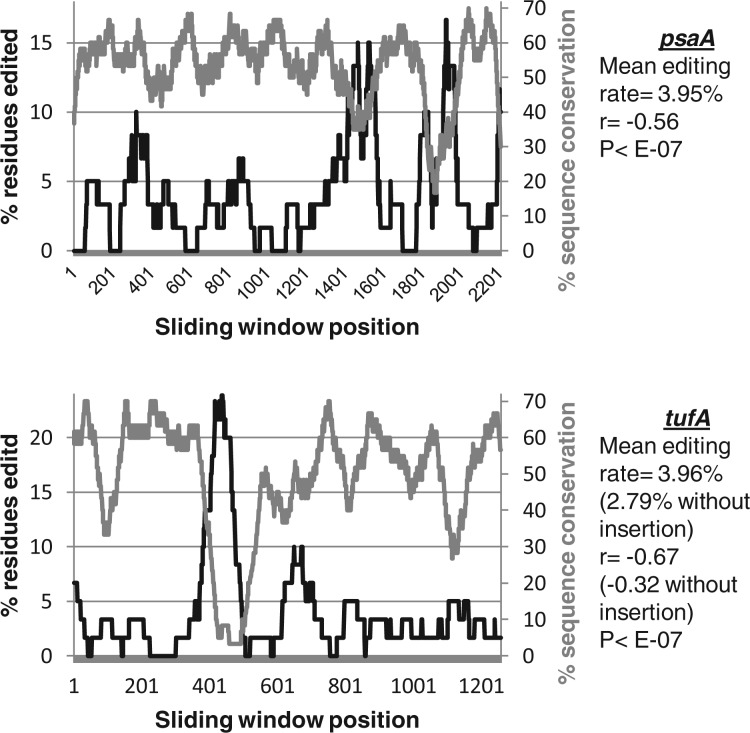
Editing is preferentially associated with highly divergent regions of *Karlodinium veneficum* plastid genes. These graphs compare the frequency of editing with sequence conservation in a 60-bp sliding window over the entire lengths of the *Karl. veneficum psaA* and *tufA* genes. The horizontal axis shows the starting position of each window within each gene sequence. The left hand vertical axis of each graph (black line) depicts the total percentage of nucleotide positions within each window that are edited within the transcript sequence. The right hand vertical axis (gray line) shows the proportion of amino acid positions within the predicted translation product of the transcript sequence of this window that are conserved with the predicted translation of the orthologous gene in the *Emiliania huxleyi* plastid. A table to the right-hand side of each graph shows the total proportion of editing sites over the entire gene, and the Pearson coefficient, and associated significance value of the correlation between sequence conservation and editing frequency. For the *tufA* gene, correlation coefficients are given both for the complete gene sequence (open figures) and for the gene sequence excluding the highly edited 84-bp insertion region specific to *Karl. veneficum* (bracketed figures). In all cases, a significant negative correlation is observed.

Certain transcripts within our data set contain highly edited regions. For example, the *psaA* and *tufA* genes both contain small regions where >15% of residues are edited, compared with an average editing rate across each gene of approximately 4% (fig. 4). To test whether these highly edited sites correspond to particularly divergent sequences, we calculated editing frequencies using a sliding 60-bp window, in polyuridylylated transcripts covering the entire *psaA* and *tufA* gene sequences. We additionally calculated the predicted sequence conservation, over the same sliding window, between the predicted *Karl. veneficum psaA* and *tufA* transcript translation products, and the corresponding *E. huxleyi* protein sequences (fig. 4). In both genes, editing was specifically correlated with low sequence conservation with *E. huxleyi* (Pearson correlation = −0.56 for *psaA,* −0.67 for *tufA*; *P* < E-07 for both genes). Notably, over a third of the editing events within *tufA* occur within an 84-bp region, which forms less than one-twelfth of the entire gene and is significantly more highly edited than the rest of the sequence (chi-squared: *P* < 0.05). This region corresponds to an in-frame insertion unique to *Karl. veneficum* (supplementary fig. S5, Supplementary Material online). Overall, our data indicate that editing events are associated with regions of sequence that are recently acquired or are highly divergent. Editing might reduce the effects of these divergent sequences on protein function.

### Editing-Facilitated Divergent C-Terminal Evolution of *Karl. veneficum* AtpA

**Fig. 5.**
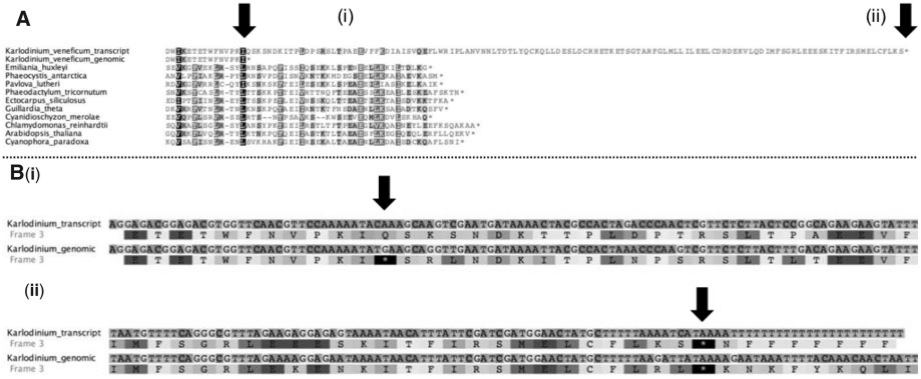
Generation of a novel C-terminal sequence extension by editing of *Karlodinium veneficum atpA* transcripts. (*A*) An alignment of the predicted translation products of the genomic and transcript sequences of *Karl. veneficum atpA* with protein sequences from other plastid lineages. (*B*) A nucleotide sequence alignment, and predicted translation products of two regions of the *Karl. veneficum* genomic and transcript sequence in detail. Residues important for defining the size of the predicted translation product of each *Karl. veneficum* sequence are labeled with vertical arrows. The *Karl. veneficum* genomic translation product terminates approximately 33-aa upstream of the consensus AtpA C-terminus, due to the presence of an in-frame TGA STOP codon within the *atpA* gene sequence. This is altered by editing to a CAA-Gln codon (*B*[*i*]) in the transcript sequence, enabling the translation of the complete AtpA C-terminus. However, the *atpA* transcript sequence is highly divergent at the 3′-end, and does not possess a termination codon at the consensus position relative to orthologous AtpA sequences. Instead, it encodes an 85-aa extension sequence that is not conserved with other AtpA sequences, which terminates in an unedited TAA STOP codon (*B*[*ii*]).

For the *Karl. veneficum atpA* gene, editing appears to be involved in the generation of a novel 3′-extension on the conventional CDS (fig. 5). The *Karl. veneficum atpA* gene contains a premature in-frame TGA codon, which is edited to form a CAA-glutamine codon in the mature transcript sequence. However, the *Karl. veneficum atpA* gene does not contain the consensus 3′-end found in other plastid sequences. The translation product of the *Karl. veneficum atpA* transcript is similar in sequence up to the final six amino acids in the *E. huxleyi* plastid AtpA protein, where it diverges to contain a 95-aa C-terminal extension that bears no homology to any other known sequence (fig. 5). The expression of this extension would be possible only from edited transcript sequence, and therefore transcript editing may have enabled divergent evolution of the ATP synthase complex in the *Karl. veneficum* plastid.

### Expression and Transcript Processing of Minicircles Located in the *Karl. veneficum* Plastid

Certain genes within the *Karl. veneficum* plastid genome, such as *rbcL* and *dnaK,* are enriched in sequencing libraries relative to others (Espelund et al. 2012). These genes have been shown not only to be encoded on the chromosomal *Karl. veneficum* plastid genome sequence but also on multiple small elements, containing fragments of individual genes, that do not assemble onto the plastid genome (Espelund et al. 2012).The episomal elements have been suggested to correspond to a population of plastid-located minicircles, which have arisen independently of those found in peridinin dinoflagellates (Zhang et al. 1999; Howe et al. 2008; Espelund et al. 2012). However, it is not known whether these episomal elements are located in the *Karl. veneficum* plastid, nor has a complete episomal element yet been sequenced and confirmed to form a minicircle.

We investigated whether episomal fragments in *Karl. veneficum* may give rise to polyuridylylated transcripts. Polyuridylylation is not found in dinoflagellate nuclei or mitochondria and would accordingly confirm localization of the elements to the *Karl. veneficum* plastid (Dorrell and Howe 2012). We initially designed primers specific to the chromosomal and episomal copies of *rbcL* and tested for the presence of polyuridylylated transcripts by oligo-d(A) primed RT-PCR, as before, but could not identify any evidence for poly(U) addition or editing on transcripts of the episomal *rbcL* elements*,* in contrast to transcripts of the chromosomal *rbcL* gene (supplementary fig. S6, Supplementary Material online).

We additionally investigated the transcription of episomal *dnaK* genes. Although there is a complete copy of the *rbcL* gene within the *Karl. veneficum* plastid genome, the chromosomal *dnaK* genes lack consensus terminal regions and contain frame-shift mutations, suggesting that they do not give rise to translationally functional *dnaK* transcripts (supplementary fig. S7*A*, Supplementary Material online) (Gabrielsen et al. 2011; Espelund et al. 2012). We could not identify polyuridylylated transcripts from either chromosomal *dnaK* gene. Instead, using PCR primers designed against different regions of *dnaK* sequence, we identified a single polyuridylylated transcript, which we term *dnaK-1* (supplementary fig. 7*A* and supplementary table S1, Supplementary Material online). The *dnaK-1* transcript encodes a complete plastid Hsp70 and does not contain any frame-shifts or align with either chromosomal *dnaK* gene, suggesting that it is expressed from an episomal element.

To identify what genetic elements might give rise to the *dnaK-1* transcript, we performed thermal asymmetric interlaced PCR (Liu et al. 1995), using combinations of primers derived from the *dnaK-1* transcript sequence. We identified a single gene that covered the entire *dnaK-1* CDS and 3′-UTR past the poly(U) site. The *dnaK-1* poly(U) site coincides with a genomic T12 motif; however, we identified *dnaK-1* transcripts through circular RT-PCR with poly(U) tails of up to 19 nt length, implying that they are generated through posttranscriptional sequence modification (supplementary fig. S7*B*, Supplementary Material online). In addition, we found evidence of extensive editing in the *dnaK-1* transcript sequence (supplementary table S2, Supplementary Material online). Overall, our data imply that *dnaK-1* is transcribed from a single contiguous genetic element, located within the *Karl. veneficum* plastid, but separate from the chromosomal genome sequence.

**Fig. 6.**
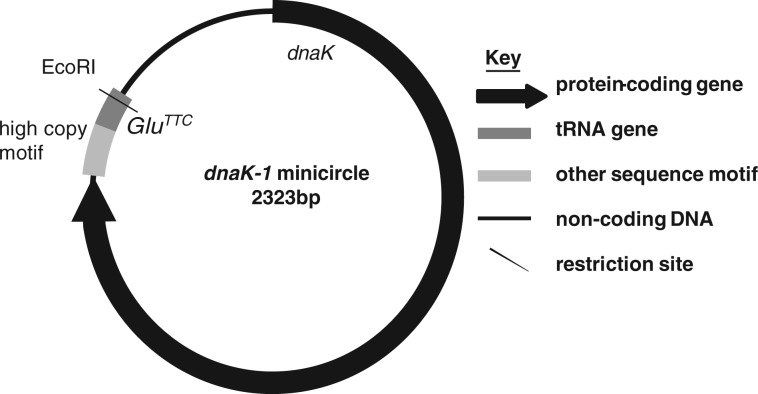
Schematic diagram of the *Karlodinium veneficum dnaK-1* minicircle. The 2,323-bp *dnaK-1* minicircle contains a complete *dnaK-1* positioned directly upstream of the predicted “high copy element,” and a *Glu^TCC^* tRNA gene in the same transcriptional orientation. A single *Eco*RI restriction site is present on the reverse strand of the tRNA gene.

Surprisingly, the *dnaK-1* 3′-UTR obtained was found to extend into a region of sequence identical to the 5′-end of the *dnaK-1* gene, consistent with the *dnaK-1* gene being located on a plastid minicircle (fig. 6). The *dnaK-1* minicircle is 2,323 bp long and contains a single *Eco*RI restriction site, which is consistent with a 2.3-kbp band containing the *dnaK* gene identified through Southern blotting of *Eco*RI-digested *Karl. veneficum* gDNA (fig. 6) (Espelund et al. 2012). In addition to a complete *dnaK* gene, this minicircle contains a *Glu^TTC^* tRNA gene, and a single “high copy” region that is conserved with other episomal sequences previously identified from *Karl. veneficum* (fig. 6) (Espelund et al. 2012). This is the first complete plastid minicircle identified in a fucoxanthin dinoflagellate, confirming that the fucoxanthin plastid genome has undergone a similar fragmentation to that observed in peridinin dinoflagellate plastid genomes. Our data furthermore show that the poly(U) and editing machinery of fucoxanthin dinoflagellates may recognize transcripts of genes encoded on minicircles over genes located on the chromosomal plastid genome.

### Absence of Poly(U) Addition and Editing from Diatom and Green Algal-Derived Serially Acquired Dinoflagellate Plastids

We wished to determine whether poly(U) addition and transcript editing are found in either dinotom or green dinoflagellate plastids, as in the fucoxanthin and peridinin-containing lineages. We performed oligo-d(A) primed RT-PCRs on five genes (*psbA, psbC, psbD, psaA,* and *rbcL*) using total cellular RNA, and PCR primers specific to the dinotom *Kryptoperidinium foliaceum* and green dinoflagellate *Lepidodinium chlorophorum* (supplementary fig. S8*A*, Supplementary Material online). We could not detect polyuridylylated transcripts for any of the genes tested (supplementary fig. S8*A*, lanes 1–5, 7–11, Supplementary Material online). We detected nonpolyuridylylated *psbA* transcripts in both species using gene-specific cDNA synthesis primers (supplementary fig. S8*A*, lanes 6 and 12, Supplementary Material online) and by circular RT-PCR (supplementary fig. S8*B*, Supplementary Material online). We could not find any evidence of editing on these transcript sequences. We conclude that poly(U) addition and editing are found only in dinoflagellates that possess the ancestral peridinin plastid or the fucoxanthin replacement lineage.

## Discussion

We have characterized the distribution and function of transcript editing and poly(U) tail addition across the entire plastid genome of the fucoxanthin dinoflagellate *Karl. veneficum*. This represents the first genome-wide study of transcript processing in a plastid acquired through serial endosymbiosis. The demonstration of poly(U) addition in *Karlodinium*, as in *Kare. mikimotoi,* indicates that it was acquired by a common ancestor of all studied fucoxanthin dinoflagellates (Bergholtz et al. 2006; Gabrielsen et al. 2011). We also found extensive sequence editing events, consistent with previous studies that identified them in both fucoxanthin dinoflagellate species (Dorrell and Howe 2012; Jackson et al. 2013). These editing events include transversion substitutions that have not previously been detected in *Karl. veneficum* but do occur in *Kare. mikimotoi*, suggesting that these are conserved across extant fucoxanthin dinoflagellates (Dorrell and Howe 2012; Jackson et al. 2013).

Many of the features associated with poly(U) addition and editing in *Karl. veneficum* have previously been documented in peridinin dinoflagellates. Multiple types of editing events have already been observed in peridinin dinoflagellates, and all species studied have had overall rates of editing of under 5%. In all species, A-G and U-C editing have been the two most abundant types of editing event (Zauner et al. 2004; Wang and Morse 2006; Dang and Green 2009; Iida et al. 2009; Mungpakdee et al. 2014). As in peridinin dinoflagellates, almost every protein-coding gene within the *Karl. veneficum* plastid can give rise to polyuridylylated transcripts, whereas tRNA genes do not possess poly(U) sites (Wang and Morse 2006; Nelson et al. 2007; Barbrook et al. 2012). Similarly, polyuridylylated polycistronic transcripts and poly(U) sites that overlap with adjacent gene sequences have previously been identified in peridinin dinoflagellates and in chromerids (Barbrook et al. 2012; Dorrell et al. 2014). This suggests that poly(U) addition has a similar functional role in transcript processing in both peridinin and fucoxanthin dinoflagellate plastids.

We also identified properties of poly(U) addition and editing that are specific to fucoxanthin dinoflagellate plastids. The editing events in *Karl. veneficum* include C-A, C-G, and U-A editing events that have not previously been detected in any peridinin dinoflagellate species although C-A editing has also been detected in *Kare. mikimotoi* (Dorrell and Howe 2012). Many of the poly(U) sites within the *Karl. veneficum* plastid are associated with housekeeping genes, which are not retained in the plastid genomes of peridinin dinoflagellates (Bachvaroff et al. 2004; Howe et al. 2008), and are plastid-located but typically do not possess poly(U) sites in chromerids (Dorrell et al. 2014).

Other unusual transcript processing features are associated with particularly divergent sequences in the *Karl. veneficum* plastid genome. The absence of poly(U) sites associated with pseudogenes has been described in chromerids (Janouskovec et al. 2013; Dorrell et al. 2014), but neither this nor a difference in the frequency of editing events on functional versus pseudogene transcripts has previously been reported in peridinin dinoflagellates. In contrast, at least some pseudogene transcripts in peridinin dinoflagellates are known to be extensively edited (Iida et al. 2009). Poly(U) addition and editing might therefore have a role in discriminating functional genes from nonfunctional gene fragments generated by recent rearrangements in fucoxanthin dinoflagellate plastid genomes. Similarly, the association of editing sites with fast-evolving sequences, such as the in-frame insertion in *tufA*, has not been described in other dinoflagellates and contrasts with plastid editing in plants, which is predominantly associated with slowly evolving sites within the genome sequence (Fujii and Small 2011; Hayes et al. 2012). These editing events might help neutralize the effects of fast-diverging sequences and recently acquired insertions on protein function.

In other cases, our data indicate that editing and poly(U) addition may have indirectly facilitated divergent sequence evolution in fucoxanthin dinoflagellate plastids. Sequence editing may have permitted the establishment of a novel 3′-sequence extension on transcripts of the *Karl. veneficum atpA* gene. To our knowledge, the edited extension of a plastid transcript into nonconserved sequence has never previously been reported. Most significantly, we have identified one plastid gene—*dnaK*—for which polyuridylylated and edited transcripts are derived from an episomal minicircle. This represents the first complete plastid minicircle sequence from a fucoxanthin dinoflagellate and suggests that the plastid genomes of fucoxanthin and peridinin dinoflagellates are undergoing convergent evolution events (Zhang et al. 1999; Espelund et al. 2012). The preferential targeting of the poly(U) and editing to *dnaK* gene copies located on minicircles may have led to their fixation over copies located on the chromosomal plastid genome, which appear to have been reduced to pseudogenes (Gabrielsen et al. 2011; Espelund et al. 2012).

Overall, our data indicate that poly(U) addition and editing in *Karl. veneficum* have evolved dynamically alongside the underlying genome, reducing the effects of mutations on plastid function, and potentially enabling the evolutionary fixation of divergent sequences. It remains to be seen whether poly(U) addition and editing were acquired after the extremely fast sequence evolution observed in the *Karl. veneficum* plastid commenced, or whether fucoxanthin plastid genomes and transcript processing have a more tightly interconnected evolutionary history. This might be resolved by investigating genome and transcriptome evolution in *Kare. mikimotoi*, or other less well-characterized fucoxanthin dinoflagellate plastids (Takishita et al. 1999; Bergholtz et al. 2006). Notably, the serially acquired plastids of dinotoms, which have less divergent genome sequences than fucoxanthin dinoflagellates, and of *Lepidodinium*, do not apply poly(U) tails or edit plastid transcripts. It will be worth determining whether the dinotoms, or *Lepidodinium,* have retained any factors involved in plastid gene expression from the ancestral peridinin symbiosis, for example by reinspecting existing transcriptome data (Minge et al. 2010; Burki et al. 2014). Further studies of dinoflagellates that have undergone serial endosymbiosis may provide important insights into the coevolution of plastid genomes and gene expression pathways.

## Materials and Methods

### Cultures

*Karlodinium veneficum* RCC2539 (also listed as UIO297) and *L. chlorophorum* (AC195) were grown in modified k/2 medium, as previously described (Dorrell and Howe 2012), under 50 μE m^−2 ^s^−1^ continuous light at a controlled temperature of 15 °C. *Kryptoperidinium* (*Glenodinium*) *foliaceum* PCC499 was grown in f/2 medium, under a 30 μE m^−2 ^s^−1^ 12:12 light:dark cycle, at 15–20 °C. To confirm the identity of the *Karl. veneficum* culture, molecular barcode sequences were generated by PCR of genomic DNA for multiple loci in the *Karl. veneficum* plastid genome. These were found to be identical to the previously published *Karl. veneficum* plastid genome sequence (strain UIO083).

### Nucleic Acid Isolation

Nucleic acids were isolated from cultures of each species harvested in early stationary phase (ca. 30–60 days postinoculation). Cells were pelleted by centrifugation and washed in sterile growth medium. For RNA isolation, 50 mg pellets of each culture were resuspended in 1 ml TRIzol reagent (Ambion), and frozen at −80 °C and thawed on ice to lyse the cells. Total cellular RNA was then isolated by phase extraction, DNase treated and cleaned with an RNeasy column (Qiagen) as previously described (Barbrook et al. 2012; Dorrell et al. 2014). Genomic DNA was isolated from cell pellets by phase extraction and cleaned with a DNeasy column as previously described (Barbrook and Howe 2000; Nash et al. 2007).

The concentration of each nucleic acid obtained was quantified using a nanodrop spectrophotometer. RNA integrity was confirmed by electrophoresis of 1 μg of each sample in an RNase-free 1% agarose gel containing 0.003% volumes of ethidium bromide. To determine whether any sample contained residual DNA contamination, each RNA sample was used as the direct substrate for a PCR using internal primers against the *psbA* gene of each sequence. Only samples for which negative results were observed in the initial PCR, and in the product of a reamplification PCR using the initial product as a PCR template, were used for further experimentation.

### RT-PCR and Sequencing

Reverse transcription was performed using Superscript III (Life Technologies), as previously described (Dorrell et al. 2014). cDNA was synthesized either with an oligo-d(A) primer, to generate products from polyuridylylated transcripts as previously described (Barbrook et al. 2012) or with internal primers specific to a particular plastid gene. PCR was performed with GoTaq flexi polymerase (Promega) as previously described (Dorrell and Howe 2012). PCR primers used are shown in supplementary table S1, Supplementary Material online. Circular RT-PCR of *Karl. veneficum* transcripts and thermal asymmetric interlaced PCR of *dnaK* genetic elements were performed as previously described (Liu et al. 1995; Dorrell and Howe 2012).

PCR products were visualized by electrophoresis in a 1% agarose-TBE gel containing ethidium bromide. Products were directly purified using a QIAquick column elution kit (Qiagen). Where multiple bands were detectable, individual products were separated by electrophoresis, cut out of the agarose gel, and purified as before. Products were sequenced using an Applied Biosystems 3730xl DNA Analyzer. The sequences of three polyuridylylated transcript sequences (*psaC, psbI*, and *psbK*) and one internal transcript sequence (*ORF4*) that were too short to be uploaded to GenBank are listed in supplementary table S2, Supplementary Material online.

### Sequence Analysis

Potential recombination events associated with the *Karl. veneficum* plastid were identified by comparison of the complete plastid genome sequence with the complete plastid genomes of the free-living haptophytes *E. huxleyi, P. globosa, Pavlova lutheri*, and the partial plastid genome of the uncultured prymnesiophyte C19487 (Puerta et al. 2005; Baurain et al. 2010; Cuvelier et al. 2010).

Poly(U) sites were identified by aligning the sequences of the oligo-d(A) RT-PCR products against the published *Karl. veneficum* plastid genome sequence (Gabrielsen et al. 2011) using GENEious (www.geneious.com). To identify motifs that might be associated with poly(U) sites, alignments were constructed of the 3′-UTR of each polyuridylylated transcript and of the first 100 bp downstream of the poly(U) site (supplementary table S2, Supplementary Material online). As a negative control, sequence alignments were constructed using the first 100 bp of the 3′-UTR sequence of each gene found not to have a poly(U) site (supplementary table S2, Supplementary Material online). The presence of primary sequence motifs that might be associated with poly(U) sites was investigated by reciprocal BLASTn searches in each alignment, and conserved RNA secondary structures were searched for using the WAR server (http://genome.ku.dk/resources/war/, last accessed June 18, 2014) (Torarinsson and Lindgreen 2008). The relative GC and purine/pyrimidine contents of each sequence were quantified using GENEious, and the minimum Gibbs free energy of folding of each sequence was calculated using the mFold server (http://mfold.rna.albany.edu, last accessed June 18, 2014) (Zuker 2003).

### Editing Analysis

Sequence editing was quantified for each gene by GENEious alignments of transcript and genomic sequences. The predicted effect of editing on protein sequence was determined by in silico translation. To determine the effect of transcript editing on protein sequence conservation between *Karl. veneficum* and haptophyte orthologs, conceptual translation sequences of the transcript and genomic sequence of each gene in the *Karl. veneficum* were aligned to plastid protein sequences from the haptophytes *E. huxleyi* and *P. globosa* using BLAST (Puerta et al. 2005). For each alignment, the number of residues conserved between the *Karl. veneficum* and haptophyte protein sequences were recorded. Identical amino acids between the two species at any position were scored as a complete match, and positives were scored as a 50% match.

To determine whether editing sites were clustered within certain regions of *Karl. veneficum* plastid genes, transcript sequences covering the entire CDS of the *psaA* and *tufA* genes were obtained by RT-PCR and aligned to the corresponding genomic sequences. Editing sites were identified in each alignment, and scored over a 60-bp sliding sequence window, and regions with elevated frequencies of editing relative to the entire CDS were identified by a binomial test. Sequence conservation between the *Karl. veneficum* and *E. huxleyi* protein sequences was scored over each window using BLAST alignment, as before. The total number of matching positions were summed over each 60-bp sliding window, and the Pearson correlation coefficients between the degree of sequence conservation and proportion of edited residues over each gene were calculated.

## Supplementary Material

Supplementary figures S1–S8 and tables S1–S4 are available at *Molecular Biology and Evolution* online (http://www.mbe.oxfordjournals.org/).

Supplementary Data

## References

[msu189-B1] Asakura Y, Bayraktar OA, Barkan A (2008). Two CRM protein subfamilies cooperate in the splicing of group IIB introns in chloroplasts. RNA.

[msu189-B2] Bachvaroff TR, Concepcion GT, Rogers CR, Herman EM, Delwiche CF (2004). Dinoflagellate expressed indicate massive transfer to the nuclear genome sequence tag data of chloroplast genes. Protist.

[msu189-B3] Barbrook AC, Dorrell RG, Burrows J, Plenderleith LJ, Nisbet RER, Howe CJ (2012). Polyuridylylation and processing of transcripts from multiple gene minicircles in chloroplasts of the dinoflagellate *Amphidinium carterae*. Plant Mol Biol..

[msu189-B4] Barbrook AC, Howe CJ (2000). Minicircular plastid DNA in the dinoflagellate *Amphidinium operculatum*. Mol Gen Genet..

[msu189-B5] Baurain D, Brinkmann H, Petersen J, Rodriguez-Ezpeleta N, Stechmann A, Demoulin V, Roger AJ, Burger G, Lang BF, Philippe H (2010). Phylogenomic evidence for separate acquisition of plastids in cryptophytes, haptophytes, and stramenopiles. Mol Biol Evol..

[msu189-B6] Bergholtz T, Daugbjerg N, Moestrup O, Fernandez-Tejedor M (2006). On the identity of *Karlodinium veneficum* and description of *Karlodinium armiger* sp nov (Dinophyceae), based on light and electron microscopy, nuclear-encoded LSU rDNA, and pigment composition. J Phycol..

[msu189-B7] Burki F, Imanian B, Hehenberger E, Hirakawa Y, Maruyama S, Keeling PJ (2014). Endosymbiotic gene transfer in tertiary plastid-containing dinoflagellates. Eukaryot Cell..

[msu189-B8] Cuvelier ML, Allen AE, Monier A, McCrow JP, Messie M, Tringe SG, Woyke T, Welsh RM, Ishoey T, Lee JH (2010). Targeted metagenomics and ecology of globally important uncultured eukaryotic phytoplankton. Proc Natl Acad Sci U S A..

[msu189-B40] Dang Y, Green BR (2009). Substitutional editing of *Heterocapsa triquetra* chloroplast transcripts and a folding model for its divergent chloroplast 16S rRNA. Gene.

[msu189-B9] Dorrell RG, Drew J, Nisbet RE, Howe CJ (2014). Evolution of chloroplast transcript processing in *Plasmodium* and its chromerid algal relatives. PLoS Genet..

[msu189-B10] Dorrell RG, Howe CJ (2012). Functional remodeling of RNA processing in replacement chloroplasts by pathways retained from their predecessors. Proc Natl Acad Sci U S A..

[msu189-B11] Espelund M, Minge MA, Gabrielsen TM, Nederbragt AJ, Shalchian-Tabrizi K, Otis C, Turmel M, Lemieux C, Jakobsen KS (2012). Genome fragmentation is not confined to the peridinin plastid in dinoflagellates. PLoS One.

[msu189-B12] Fujii S, Small I (2011). The evolution of RNA editing and pentatricopeptide repeat genes. New Phytol..

[msu189-B13] Gabrielsen TM, Minge MA, Espelund M, Tooming-Klunderud A, Patil V, Nederbragt AJ, Otis C, Turmel M, Shalchian-Tabrizi K, Lemieux C (2011). Genome evolution of a tertiary dinoflagellate plastid. PLoS One.

[msu189-B14] Green BR (2011). Chloroplast genomes of photosynthetic eukaryotes. Plant J..

[msu189-B15] Hayes ML, Giang K, Mulligan RM (2012). Molecular evolution of pentatricopeptide repeat genes reveals truncation in species lacking an editing target and structural domains under distinct selective pressures. BMC Evol Biol..

[msu189-B16] Howe CJ, Nisbet RER, Barbrook AC (2008). The remarkable chloroplast genome of dinoflagellates. J Exp Bot..

[msu189-B17] Iida S, Kobiyama A, Ogata T, Murakami A (2009). Identification of transcribed and persistent variants of the *psbA* gene carried by plastid minicircles in a dinoflagellate. Curr Genet..

[msu189-B18] Imanian B, Pombert JF, Keeling PJ (2010). The complete plastid genomes of the two ‘dinotoms’ *Durinskia baltica* and *Kryptoperidinium foliaceum*. PLoS One.

[msu189-B19] Jackson CJ, Gornik SG, Waller RF (2013). A tertiary plastid gains RNA editing in its new host. Mol Biol Evol..

[msu189-B20] Janouskovec J, Horák A, Oborník M, Lukes J, Keeling PJ (2010). A common red algal origin of the apicomplexan, dinoflagellate, and heterokont plastids. Proc Natl Acad Sci U S A..

[msu189-B21] Janouskovec J, Sobotka R, Lai DH, Flegontov P, Koník P, Komenda J, Ali S, Prásil O, Pain A, Oborník M (2013). Split photosystem protein, linear-mapping topology, and growth of structural complexity in the plastid genome of *Chromera velia*. Mol Biol Evol..

[msu189-B22] Lange H, Sement FM, Canaday J, Gagliardi D (2009). Polyadenylation-assisted RNA degradation processes in plants. Trends Plant Sci..

[msu189-B23] Larson EM, Obrien CM, Zhu GH, Spreitzer RJ, Portis AR (1997). Specificity for activase is changed by a Pro-89 to Arg substitution in the large subunit of ribulose-1,5-bisphosphate carboxylase/oxygenase. J Biol Chem..

[msu189-B24] Li CH, Salvucci ME, Portis AR (2005). Two residues of rubisco activase involved in recognition of the rubisco substrate. J Biol Chem..

[msu189-B25] Liu YG, Mitsukawa N, Oosumi T, Whittier RF (1995). Efficient isolation and mapping of *Arabidopsis thaliana* T-DNA insert junctions by thermal asymmetric interlaced PCR. Plant J..

[msu189-B26] Matsumoto T, Shinozaki F, Chikuni T, Yabuki A, Takishita K, Kawachi M, Nakayama T, Inouye I, Hashimoto T, Inagaki Y (2011). Green-colored plastids in the dinoflagellate genus *Lepidodinium* are of core chlorophyte origin. Protist.

[msu189-B27] Minge MA, Shalchian-Tabrizi K, Torresen OK, Takishita K, Probert I, Inagaki Y, Klaveness D, Jakobsen KS (2010). A phylogenetic mosaic plastid proteome and unusual plastid-targeting signals in the green-colored dinoflagellate *Lepidodinium chlorophorum*. BMC Evol Biol..

[msu189-B41] Mungpakdee S, Shinzato C, Takeuchi T, Kawashima T, Koyanagi R, Hisata K, Tanaka M, Goto H, Fujie M, Lin S (2014). Massive gene transfer and extensive RNA editing of a symbiotic dinoflagellate plastid genome. Genom Biol Evol..

[msu189-B28] Nash EA, Barbrook AC, Edwards-Stuart RK, Bernhardt K, Howe CJ, Nisbet RER (2007). Organization of the mitochondrial genome in the dinoflagellate *Amphidinium carterae*. Mol Biol Evol..

[msu189-B29] Nelson MJ, Dang YK, Filek E, Zhang ZD, Yu VWC, Ishida K, Green BR (2007). Identification and transcription of transfer RNA genes in dinoflagellate plastid minicircles. Gene.

[msu189-B30] Puerta MVS, Bachvaroff TR, Delwiche CF (2005). The complete plastid genome sequence of the haptophyte *Emiliania huxleyi*: a comparison to other plastid genomes. DNA Res..

[msu189-B31] Shalchian-Tabrizi K, Skanseng M, Ronquist F, Klaveness D, Bachvaroff TR, Delwiche CF, Botnen A, Tengs T, Jakobsen KS (2006). Heterotachy processes in rhodophyte-derived secondhand plastid genes: implications for addressing the origin and evolution of dinoflagellate plastids. Mol Biol Evol..

[msu189-B32] Takano Y, Hansen G, Fujita D, Horiguchi T (2008). Serial replacement of diatom endosymbionts in two freshwater dinoflagenates, *Peridiniopsis* spp. (Peridiniales, Dinophyceae). Phycologia.

[msu189-B33] Takishita K, Kawachi M, Noel M-H, Matsumoto T, Kakizoe N, Watanabe MM, Inouye I, Ishida K-I, Hashimoto T, Inagaki Y (2008). Origins of plastids and glyceraldehyde-3-phosphate dehydrogenase genes in the green-colored dinoflagellate *Lepidodinium chlorophorum*. Gene.

[msu189-B34] Takishita K, Nakano K, Uchida A (1999). Preliminary phylogenetic analysis of plastid-encoded genes from an anomalously pigmented dinoflagellate *Gymnodinium mikimotoi* (Gymnodiniales, Dinophyta). Phycol Res..

[msu189-B35] Torarinsson E, Lindgreen S (2008). WAR: Webserver for aligning structural RNAs. Nucl Acids Res..

[msu189-B36] Wang YL, Morse D (2006). Rampant polyuridylylation of plastid gene transcripts in the dinoflagellate *Lingulodinium*. Nucl Acids Res..

[msu189-B37] Zauner S, Greilinger D, Laatsch T, Kowallik KV, Maier UG (2004). Substitutional editing of transcripts from genes of cyanobacterial origin in the dinoflagellate *Ceratium horridum*. FEBS Lett..

[msu189-B38] Zhang Z, Green BR, Cavalier-Smith T (1999). Single gene circles in dinoflagellate chloroplast genomes. Nature.

[msu189-B39] Zuker M (2003). Mfold web server for nucleic acid folding and hybridization prediction. Nucl Acids Res..

