# Supplementary material for: Genome-Wide Transcript Profiling Reveals the Coevolution of Plastid Gene Sequences and Transcript Processing Pathways in the Fucoxanthin Dinoflagellate Karlodinium veneficum
Source: Mol Biol Evol. 2014 Jun 12;31(9):2376–86. doi: 10.1093/molbev/msu189 (PMC4137713; doi:10.1093/molbev/msu189)
Supplement: Supplementary Data [file supp_31_9_2376__index.html]

Genome-wide transcript profiling reveals the coevolution of plastid gene sequences and transcript processing pathways in the fucoxanthin dinoflagellate Karlodinium veneficum — Genome-Wide Transcript Profiling Reveals the Coevolution of Plastid Gene Sequences and Transcript Processing Pathways in the Fucoxanthin Dinoflagellate Karlodinium veneficum — Genome-Wide Transcript Profiling Reveals the Coevolution of Plastid Gene Sequences and Transcript Processing Pathways in the Fucoxanthin Dinoflagellate Karlodinium veneficum — Supplementary Data 

# Genome-Wide Transcript Profiling Reveals the Coevolution of Plastid Gene Sequences and Transcript Processing Pathways in the Fucoxanthin Dinoflagellate *Karlodinium veneficum*

## Supplementary Data

files

**Files in this Data Supplement:**

- Supplementary Data - pdf file
- Supplementary Data - xlsx file
- Supplementary Data - xlsx file
- Supplementary Data - xlsx file
- Supplementary Data - xlsx file
