# Supplementary material for: Genome-Wide Transcript Profiling Reveals the Coevolution of Plastid Gene Sequences and Transcript Processing Pathways in the Fucoxanthin Dinoflagellate Karlodinium veneficum
Source: Mol Biol Evol. 2014 Jun 12;31(9):2376–86. doi: 10.1093/molbev/msu189 (PMC4137713; doi:10.1093/molbev/msu189)
Supplement: Supplementary Data [file supp_msu189_Supplementary_figures_and_table_legends.pdf]

Article: Discoveries

Genome-wide transcript profiling reveals the coevolution of plastid gene sequences and transcript processing pathways in the fucoxanthin dinoflagellate *Karlodinium veneficum*

Supporting figures and table legends

Elisabeth Richardson*[1], Richard. G. Dorrell[1]*, Christopher .J. Howe+[1]

[1]Department of Biochemistry, University of Cambridge, Building O, Downing Site, Tennis Court Road, Cambridge, CB2 1QW UK

*contributed equally to this work

+to whom correspondence should be addressed: ch26@cam.ac.uk

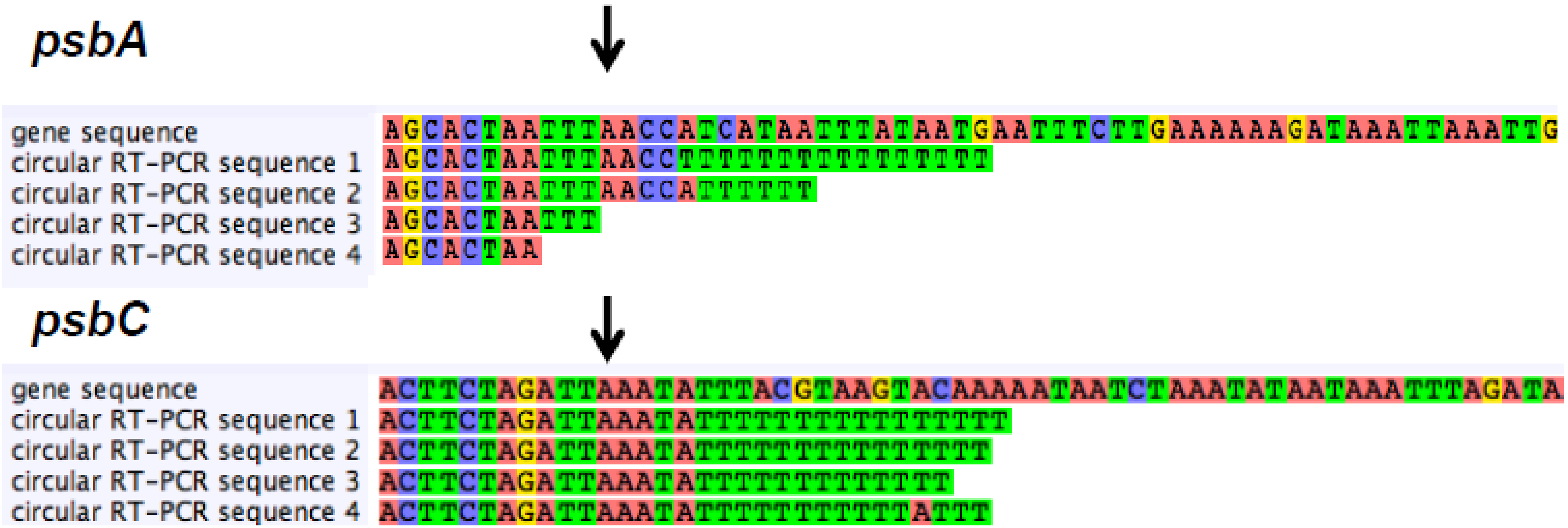

Fig. S1. Circular RT-PCRs of *Karlodinium veneficum psbA* and *psbC* transcripts. This figure shows the 3' termini of *psbA* and *psbC* transcripts, as identified by circular RT-PCR, aligned with the corresponding genomic sequences. For each gene, the final 10 nt of coding sequence and the first 50 nt of the 3' UTR is shown. A vertical arrow corresponds to the TAA-STOP codon employed by each gene. For both genes, transcripts could be identified that clearly terminated at the 3' end in the 3' UTR, and possessed a poly(T) sequence that did not correspond to the underlying genomic sequence, confirming that transcripts in the *K. veneficum* plastid receive a post-transcriptional 3' poly(U) tail. All of the poly(U) tails identified were homopolymeric, with the exception of one, associated with a *psbA* transcript, which contained a single adenosine residue. Although two of the *psbA* transcripts identified did not possess a 3' poly(U) tail, these transcripts terminate within the CDS, upstream of the STOP codon, and are therefore likely to represent the degradation products of polyuridylylated transcripts.

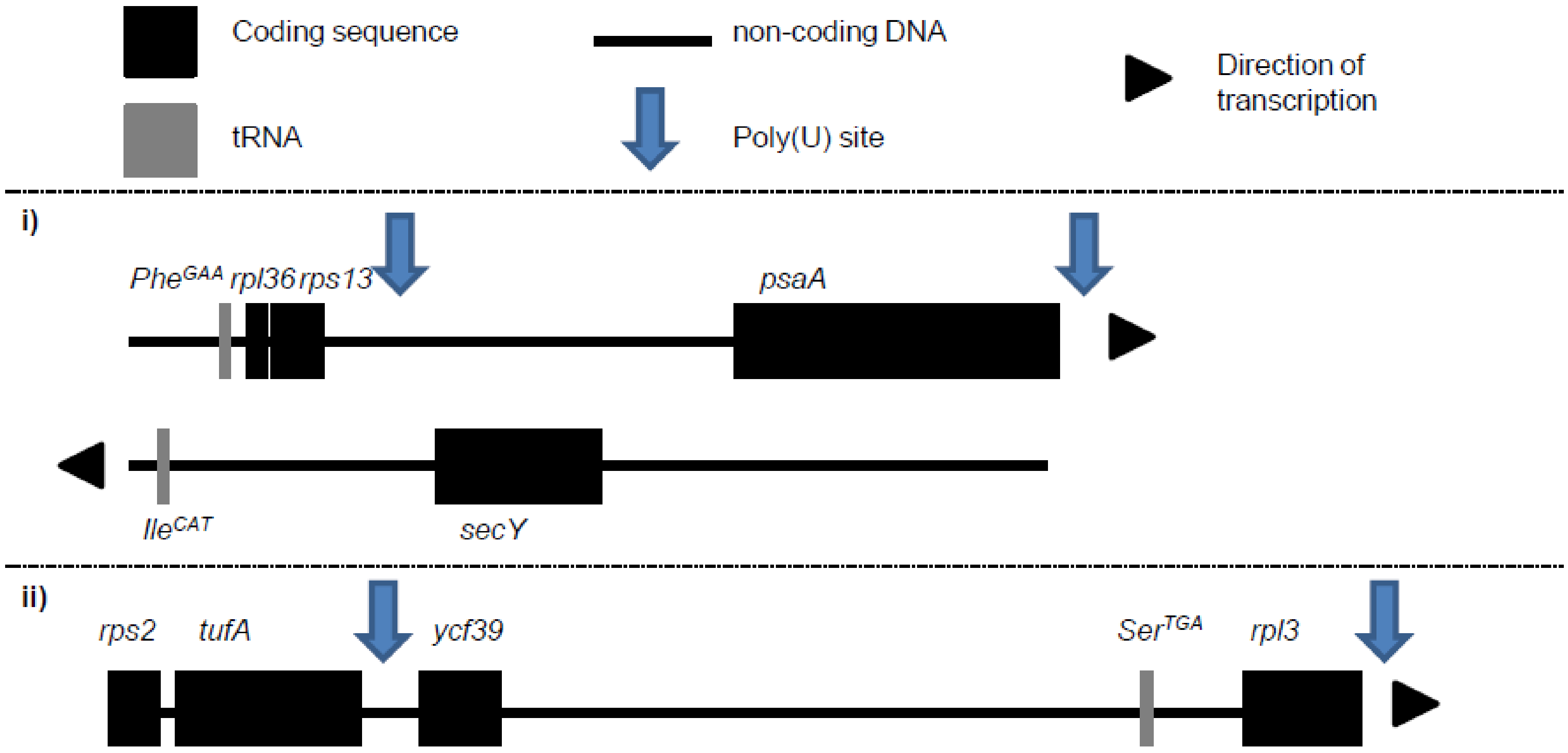

Fig. S2. Genomic context of non-polyuridylylated protein-coding genes in the *Karlodinium veneficum* plastid. These diagrams show the order of genes surrounding, *secY* (i) and *ycf39* (ii), neither of which give rise to polyuridylylated transcripts, in the *K. veneficum* plastid. The coding content of each strand is shown separately. *secY* is located between two genes (*rps13, psaA*) of opposing transcriptional orientation, and is located over 15kbp upstream of the *psaF-psbJ* fusion gene poly(U) site. *ycf39* is located immediately upstream of a large region of DNA with no annotated function, and the nearest poly(U) site (associated with *rpl3*) is nearly 6kbp downstream.

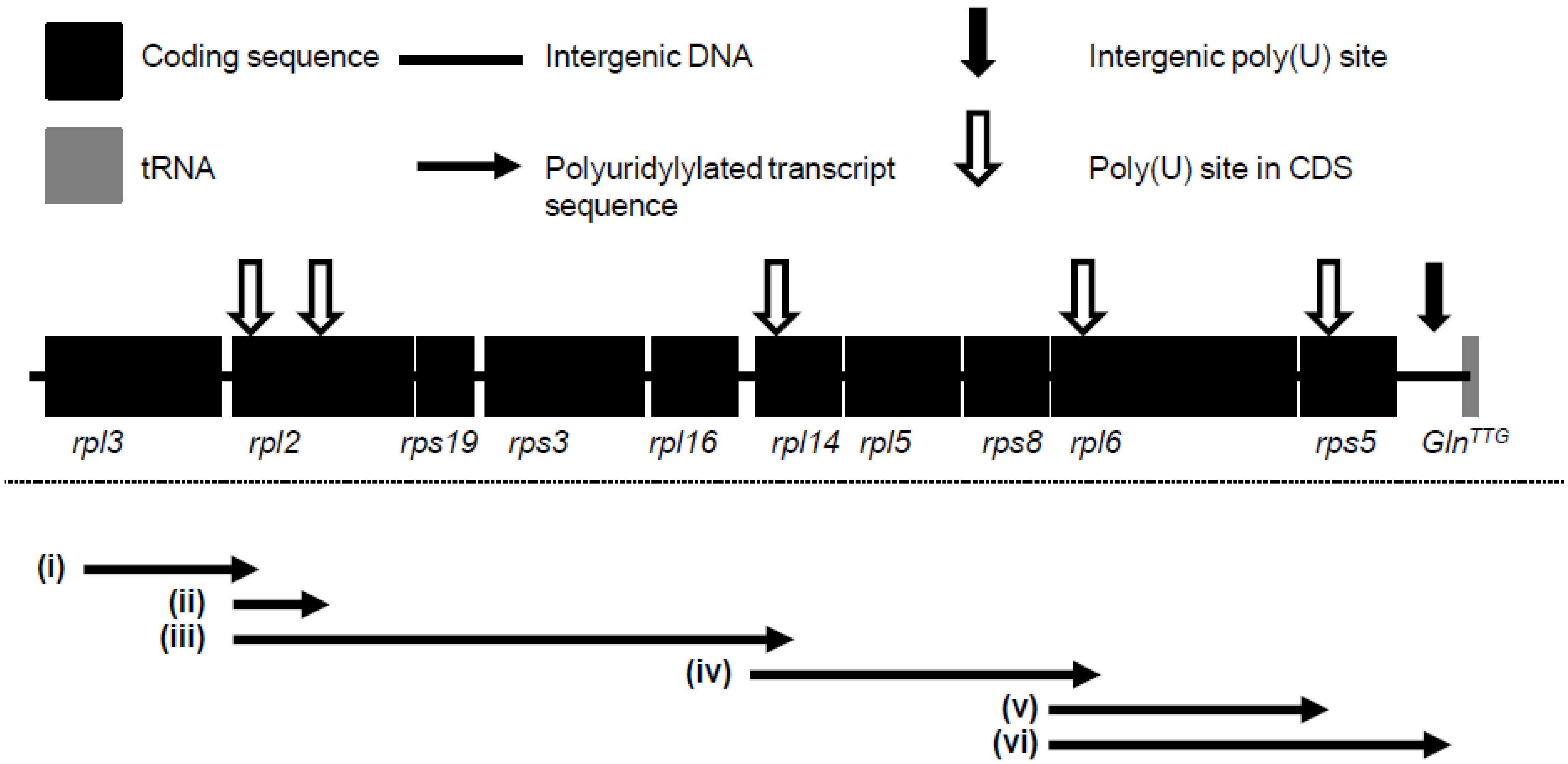

Fig. S3. Overlapping poly(U) sites within the *Karlodinium veneficum* ribosomal protein superoperon. This diagram shows the array of 3' termini associated with polyuridylylated transcripts, identified by oligo-d(A) primed RT-PCR over ten genes extending from *rpl3* to *rps5* in the *K. veneficum* plastid genome. (i), *rpl3* transcript with poly(U) site positioned in *rpl2*; (ii), internally polyuridylylated *rpl2* transcript; (iii), polycistronic polyuridylylated *rpl2-rps19-rps3-rpl16* transcript with poly(U) site positioned in *rpl14*; (iv), polycistronic polyuridylylated *rpl14-rpl5-rps8* transcript with poly(U) site positioned in *rpl6*; (v), polyuridylylated *rpl6* transcript with poly(U) site positioned in *rps5*; and (vi), polycistronic polyuridylylated *rpl6-rps5* transcript.

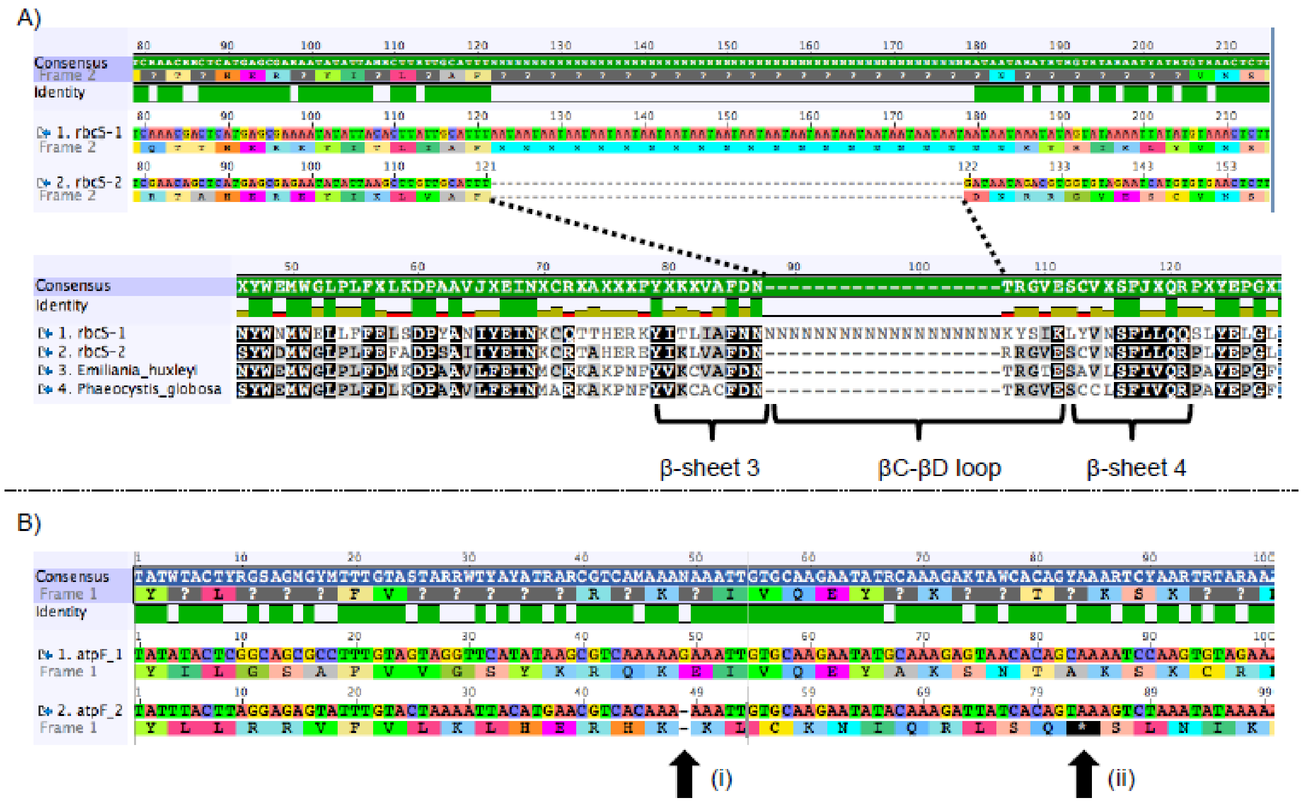

Fig. S4. Aligned protein and transcript sequences of paralogous copies of the *rbcS* and *atpF-2* sequences in the *Karlodinium veneficum* plastid. Panel A shows the aligned sequences of *rbcS-1* and *rbcS-2*, as sequenced from RT-PCR products generated with a gene-specific internal cDNA primer. The *rbcS-1* transcript contains a 66 bp poly(AAT) insertion, which would be translated in-frame as a poly(N) sequence. This sequence is not found in orthologous copies of the rbcS protein sequence from the representative free-living haptophytes *Emiliania huxleyi* and *Phaeocystis globosa*, and is predicted to be positioned immediately downstream of β-sheet 3, extending into the βC-βD loop domain in the *K. veneficum* RbcS-1 protein sequence. The expression product of *rbcS-2*, by contrast, aligns well with the haptophyte RbcS sequences. Panel B shows the aligned sequences of *K. veneficum atpF-1* and *atpF-2*, as obtained from RT-PCR products as before. The *atpF-1* and *atpF-2* sequences are similar to each other, except in the presence of a single nucleotide deletion (i) in the *atpF-2* transcript. This causes a sequence frame-shift that is predicted to lead to the translation of a premature termination codon located 36 bp downstream of the deletion site (ii), preventing the expression of a full-length, functional atpF protein sequence from *atpF-2*.

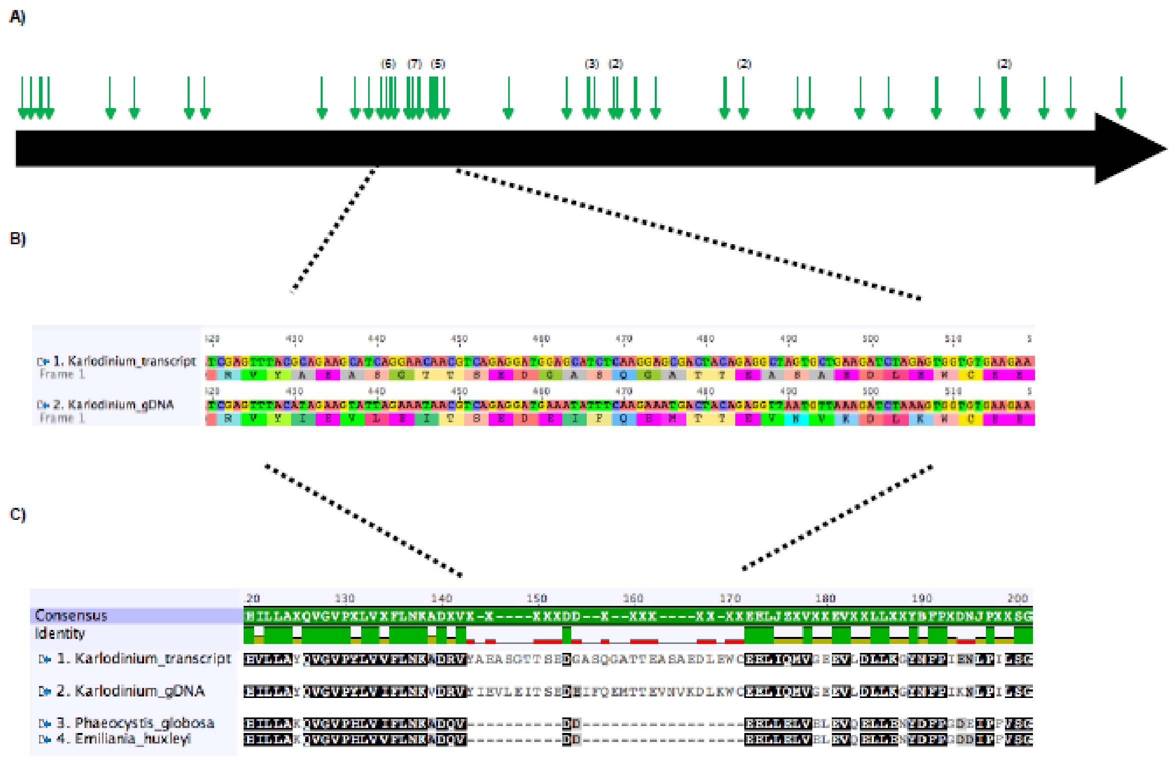

Fig. S5. Editing of a novel in-frame insertion in *Karlodinium veneficum tufA*. These alignments show the unusually high levels of editing associated with an internal in-frame insertion sequence unique to *K. veneficum tufA*. Panel A shows a schematic diagram of the entire *K. veneficum tufA* gene. Sites that are edited in the transcript sequences are labelled, to scale, with vertical green arrows. In certain positions, editing events are so tightly clustered as to prevent the annotation of each site independently, and for these the total number of editing events are given in brackets above the alignment.

One particular region of the *K. veneficum tufA* sequence is highly edited. The nucleotide sequence and predicted translation products of the genomic versus transcript sequences over this region are shown in panel B. In total, 18 separate editing events are observed over an 84 bp region of sequence, many of which are predicted to have non-synonymous effects on the eventual translation product. The translation products of this region, and the sequences that surround it, are aligned against representative haptophyte TufA protein sequences in panel C. The highly edited 84 bp region is found to correspond to a novel, in-frame 28aa insertion, positioned downstream of residues 142-144 in the orthologous *E. huxleyi* TufA sequence.

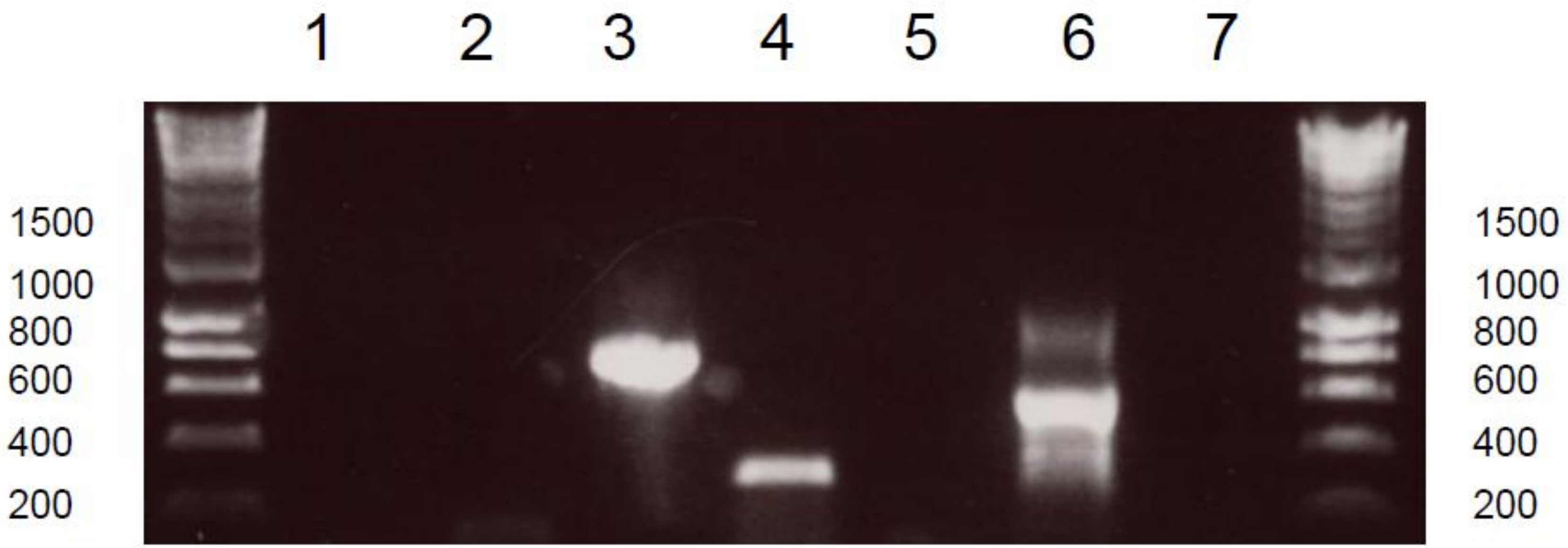

Fig. S6. Absence of polyuridylylated transcripts from episomal fragment copies of the *Karlodinium veneficum rbcL* gene. This gel photo shows the result of a series of RT-PCRs to detect poly(U) tails on copies of *rbcL* located either on the chromosomal plastid genome, or separate episomal elements in the *K. veneficum* genome. Lane 1-2: oligo-d(A) RT-PCR using forward primers specific to two episomal *rbcL* sequences (*rbcL* fragments 1, 2; GBID: 185572.1, 185573.1), demonstrating the absence of polyuridylylated transcripts of either gene. Lane 3: oligo-d(A) RT-PCR using a forward primer specific to the complete *rbcL* gene contained in the chromosomal plastid genome, confirming the presence of polyuridylylated transcripts. Lanes 4-5: RT-PCR of *rbcL* fragment 1 using a gene-specific cDNA synthesis primer, under template positive and negative conditions, and lanes 6-7: RT-PCR of *rbcL* fragment 2 using a gene-specific cDNA synthesis primer, under template positive and negative conditions, confirming the presence of non-polyuridylylated transcripts of each episomal fragment. In total, there was only a single nucleotide difference over the 878 bp non-polyuridylylated transcript sequences amplified relative to the underlying genomic sequences, suggesting that little editing occurs.

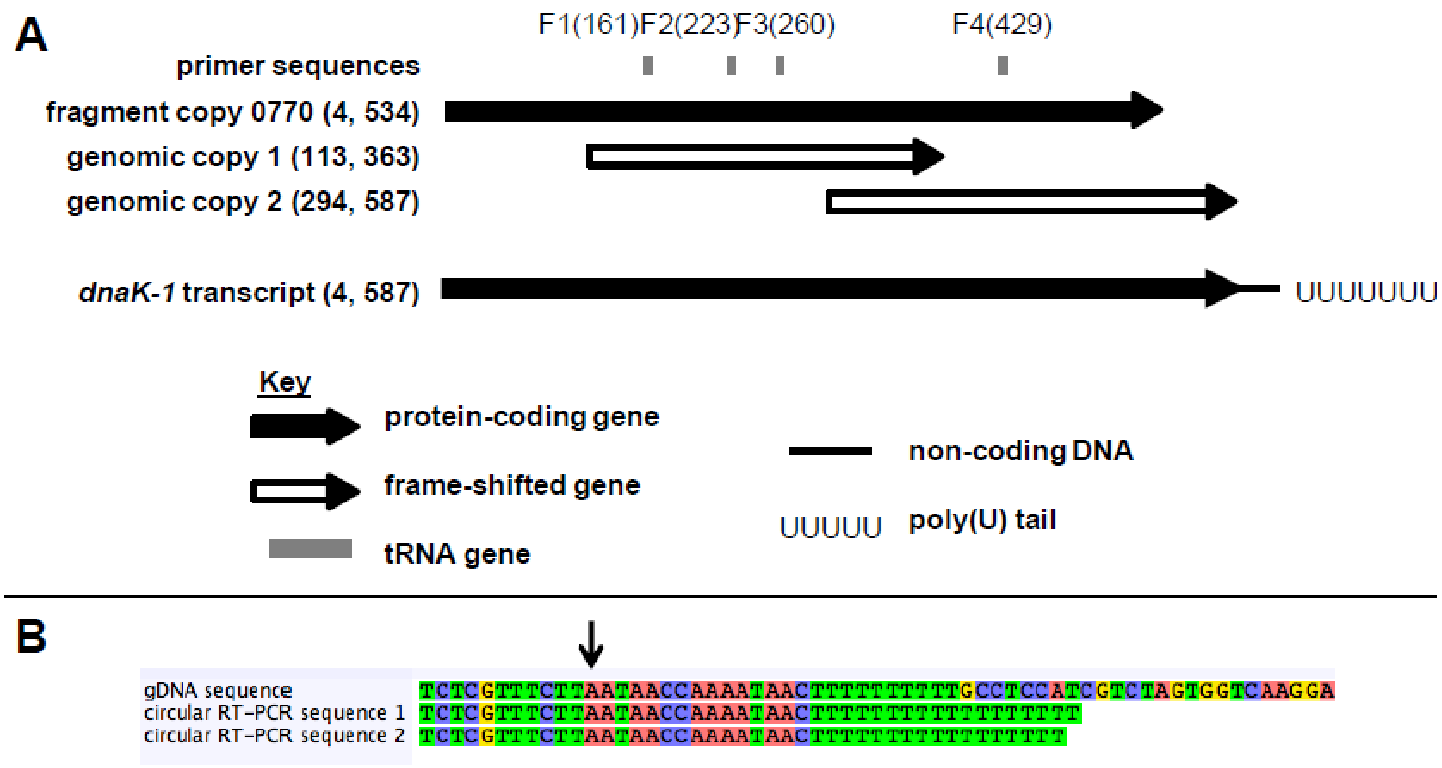

Fig. S7. Alignments of genetic and transcript *Karlodinium veneficum dnaK* sequences. Panel A shows an alignment of the two copies of *dnaK* located on the *K. veneficum* plastid genome, the extensive copy of *dnaK* located on episomal fragment 0770, the four primers generated from this sequence to perform oligo-d(A) RT-PCR, and the complete *dnaK-1* CDS. Numbers in brackets after each gene name correspond to the beginning and end of the regions of the *E. huxleyi* plastid Hsp70 protein sequence to which each gene product is homologous. Bracketed numbers after each primer correspond to the equivalent position of the primer 3' end on the *E. huxleyi* protein sequence.

Panel B shows an alignment of the 3' termini of *dnaK-1* transcripts as obtained by circular RT-PCR with the underlying genomic sequence, displayed as in fig. S1. Although the *dnaK-1* poly(U) site corresponds to a poly(T) tract in the genomic sequence, this is shorter (12 nucleotides) than the poly(U) tails identified through circular RT-PCR (18-19 nucleotides). This demonstrates that the *dnaK-1* transcripts are subject to post-transcriptional 3' poly(U) tail addition, confirming their localisation to the *K. veneficum* plastid.

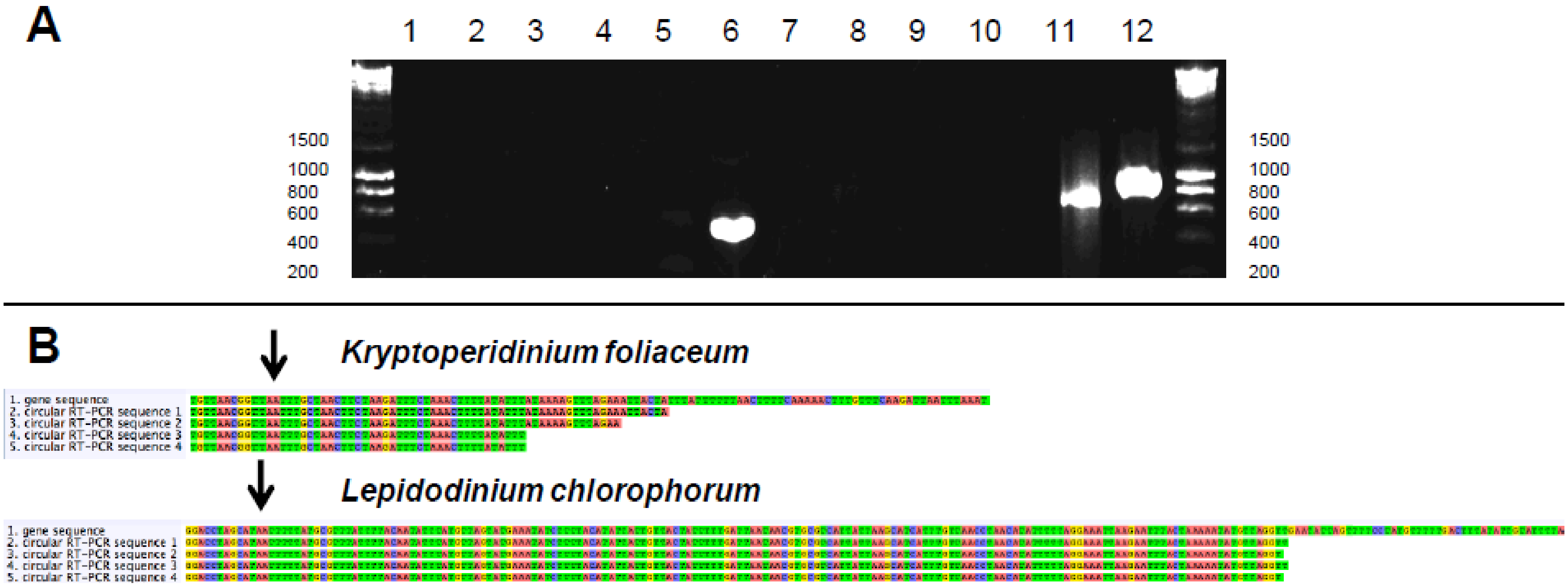

Fig. S8. Poly(U) addition is found only in fucoxanthin-containing serial dinoflagellate plastids. Panel A shows a gel photo for a series of oligo-d(A) RT-PCRs to detect polyuridylylated transcripts in representative dinotom *(Kryptoperidinium foliaceum*) and green dinoflagellate *(Lepidodinium chlorophorum*) plastids. Lanes 1-5: oligo-d(A) RT-PCRs of *K. foliaceum psbA, psbC, psbD, psaA, rbcL*, and lanes 6-10: oligo-d(A) RT-PCRs of *L. chlorophorum psbA, psbC, psbD, psaA, rbcL.* The absence of products from lanes 1-4, 6-10, indicates that polyuridylylated transcripts of each gene are not present. The same results were observed when the primary product for each reaction was used as template for an additional 40 cycles of PCR amplification. The RT-PCR product from lane 5 was sequenced, and found to be a PCR chimera. Lane 6: RT-PCR of *K. foliaceum psbA* with an internal gene-specific cDNA synthesis primer, and the same PCR forward primer as in lane 1, and lane 12: RT-PCR of *L. chlorophorum psbA* with an internal gene-specific cDNA synthesis primer, and the same PCR forward primer as in lane 7, confirming the presence of transcripts of each gene in the corresponding RNA sample, and demonstrating that the absence of products from the corresponding oligo-d(A) primed RT-PCR was not due to failure of the PCR primer to anneal to the desired template sequence. The RT-PCR products from lanes 6 and 12 were sequenced, and compared to PCR products amplified from gDNA from each species, and were not found to contain

any putative editing sites, suggesting that they were derived from non-polyuridylylated and unedited transcript sequences. Lane 13: oligo-d(A) RT-PCR of *Karlodinium veneficum psbA*, forming a positive control for polyuridylylated and edited transcripts.

Panel B shows the aligned 3' termini of *Kryptoperidinium foliaceum* and *Lepidodinium chlorophorum psbA* transcript sequences, as identified by circular RT-PCR, with the underlying genomic sequence as amplified by TAiL-PCR, displayed as in fig. S1. In each case, the transcript sequences identified terminate within the 3' UTR of the gene, without a poly(U) tail or any other form of 3' terminal modification. This confirms that mature transcripts in dinotom and green dinoflagellate plastids do not receive poly(U) tails.

Supplementary Tables

Table S1. Tabulated primers used.

Table S2. Total transcriptome data from the *Karlodinium veneficum* plastid. Panel A shows the position of poly(U) sites and extent of editing across every gene in the *K. veneficum* plastid. Genes are separated into those that possess poly(U) sites in the 3' UTR, and those that do not. For polyuridylylated genes, the associated 3' UTR sequence, and the first 100 bp of sequence downstream of the poly(U) site, used to search for conserved poly(U) associated motifs, are tabulated. For genes that lack-poly(U) sites, the total 3' UTR sequence is recorded, along with whether transcripts of the gene could be detected as part of polyuridylylated polycistronic transcripts, and the immediate genes located downstream. (anti) indicates that the downstream gene is in a reverse transcriptional orientation. Editing sites for the CDS and 3' UTR of each gene are listed separately. Panel B contains the sequence of three polyuridylylated transcripts (*psaC, psbI, psbK*) that were too short to be uploaded to GenBank.

Table S3. Sequence evolution of genes that possess unusual poly(U) sites in the *Karlodinium veneficum* plastid. This table lists all of the genes that lack poly(U) sites, or possess overlapping poly(U) sites, in the *K. veneficum* plastid genome. The genes positioned downstream of each gene are listed for *K. veneficum*, and for four representative haptophyte plastid genome sequences (*Emiliania huxleyi, Phaeocystis globosa, Pavlova lutheri,* and the uncultured prymnesiophyte C19847). The genes positioned upstream of *psbN, secY* and *ycf39*, which could not be assembled onto any polyuridylylated transcripts, are also tabulated for each species. Sequence recombination and deletion events that can be inferred from this data as having occurred in an early ancestor of *K. veneficum* are listed in the right-hand column. Although some genes that lack poly(U) sites in *K. veneficum* are located upstream of likely recombination sites (*psbF, psbN, secY rps11, ycf3*) or gene deletion events (*rps9, rps19*) (Table S3), many others are not.

Table S4. Effect of editing on the predicted translation products of transcript sequences from the *Karlodinium veneficum* plastid. Panel A lists the in-frame termination codons present in eleven genes within the *Karlodinium* plastid genome, and the codon in each corresponding transcript sequence. In each case, editing of the transcript sequence alters the termination codon to one of coding function, enabling translation of the complete transcript

sequence. Panels B-D list the total degree of sequence conservation, as determined by BLASTx alignments, of the predicted translation products of transcript and genomic sequences from the *K. veneficum* plastid against two representative haptophyte lineages *(Emiliania huxleyi, Phaeocystis globosa)*. Editing broadly increases the degree of protein sequence conservation with each reference dataset, suggesting that editing is involved in correcting divergent codons in the transcript sequence.
